# Intestinal Microecology Dysbiosis, Cultivable Pathogen Composition, and Antimicrobial Susceptibility Profiles Associated with Intestinal Exposure and Ocular Opacity in Hybrid Porcupinefish

**DOI:** 10.3390/microorganisms14081703

**Published:** 2026-08-03

**Authors:** Xiaojie Bai, Zuoping Xie, Xiangyu Du, Fangyan Jiang, Ning Yang, Hai Huang

**Affiliations:** 1 Yazhou Bay Innovation Institute, Hainan Tropical Ocean University, Sanya 572022, China; b17733266464@163.com (X.B.);; 2 Key Laboratory of Utilization and Conservation for Tropical Marine Bioresources of Ministry of Education, Hainan Tropical Ocean University, Sanya 572022, China; 3 Hainan Key Laboratory for Conservation and Utilization of Tropical Marine Fishery Resources, Hainan Tropical Ocean University, Sanya 572022, China; 4 Tropical Biodiversity and Bioresource Utilization Laboratory, Qiongtai Normal University, Haikou 571127, China

**Keywords:** hybrid porcupinefish, intestinal exposure, corneal opacity, intestinal microbiota, *Vibrio harveyi*, antimicrobial susceptibility

## Abstract

Intestinal exposure and ocular opacity occur in cultured hybrid porcupinefish, but the associated pathological lesions and gut microecological changes remain poorly characterized. Hybrid porcupinefish were assigned to a healthy control group (CT-CD-JK), an intestinal exposure group (CT-CD-FB), or an ocular opacity group (CT-YQ-FB). Gut microbiota, histopathology, cultivable bacteria, and antimicrobial susceptibility were assessed using 16S rRNA sequencing, hematoxylin and eosin (H&E) staining, bacterial isolation and identification, and disk diffusion testing. Diseased fish showed evident intestinal and ocular tissue injury. Microbial richness was reduced in the CT-CD-FB group, and gut microbial community structure differed among groups (permutational multivariate analysis of variance [PERMANOVA], R^2^ = 0.5058, *p* = 0.001). At the phylum level, Firmicutes increased in the CT-CD-FB group, whereas Proteobacteria accounted for a higher proportion in the CT-CD-JK group. Linear discriminant analysis effect size (LEfSe) further indicated group-specific enrichment patterns. A total of 23 cultivable isolates were obtained, among which isolates assigned as putative *Vibrio harveyi* based on 16S rRNA sequence alignment accounted for 90.9% of isolates derived from intestinal contents in the CT-CD-FB group, which was higher than the 16.7% observed in the CT-CD-JK group. Antimicrobial susceptibility testing suggested a potential multidrug-resistance risk among the isolates; however, higher proportions of susceptible (S) isolates were observed for ceftriaxone (CTR), quinolones, aminoglycosides, and chloramphenicol (C). These preliminary findings suggest that gut dysbiosis, intestinal tissue injury, and a higher detection proportion of putative *Vibrio harveyi* were associated with intestinal exposure in this small cohort of naturally diseased hybrid porcupinefish, providing a basis for subsequent pathogen-verification studies and antimicrobial-susceptibility assessment.

## 1. Introduction

The rapid development of the aquaculture industry has improved the supply capacity of high-quality animal protein, but high-density farming, environmental fluctuations, and pathogen transmission have also increased the risk of bacterial disease outbreaks in farmed fish. Vibriosis is one of the most important bacterial diseases in marine fish aquaculture, frequently associated with high mortality and significant economic losses, and can manifest clinically as skin ulceration, septicemia, gastroenteritis, ocular injury, and muscle necrosis [1,2]. In the culture of tetraodontiformes and related commercially important marine fish, environmental stress and pathogen infection often exhibit a superimposed relationship; for example, under the combined stress of hypoxia and *Vibrio parahaemolyticus*, *Takifugu obscurus* can show host responses related to oxidative stress and inflammation, accompanied by a higher risk of adverse outcomes [3]. These pieces of evidence suggest that diseases in cultured fish are often not driven by a single factor, but may instead be the comprehensive result of co-alterations in host immune status, environmental stress, intestinal barrier function, and microbial communities. These findings suggest that diseases in cultured fish are often multifactorial, involving host immunity, environmental stress, intestinal barrier function, and microbial communities. Clinical observation alone is therefore insufficient to explain intestinal exposure and ocular opacity in hybrid porcupinefish, and additional evidence is needed to clarify potential etiological factors.

The fish intestinal microbiota is considered to be closely associated with host nutrient absorption, immune homeostasis, mucosal barrier maintenance, and disease resistance. Recent reviews point out that the fish intestinal microbial community is influenced by multiple factors such as host species, feed composition, developmental stage, salinity, water temperature, water quality, and farming methods, with common dominant phyla including Proteobacteria, Firmicutes, Bacteroidetes, and Actinobacteria [4,5]. Under different environmental pressures and management conditions, the structure and functional potential of the intestinal microbiota may undergo adaptive changes, which correlate with host immune regulation and barrier integrity [5,6]. In hybrid fish, the intestinal microbiota may also be co-shaped by the hybrid background and culture conditions, presenting more obvious individual differences and plasticity [7]. When fish are affected by pathogen infection or adverse environmental factors, intestinal dysbiosis may occur, manifesting as decreased community diversity, rearrangement of dominant taxa, and an increased proportion of potential pathogenic bacteria, which is associated with changes in health outcomes [6]. For example, rainbow trout can experience persistent intestinal microbiota changes after infection and antibiotic treatment, suggesting that infection-related events can exert a long-term impact on the digestive tract microecology of cultured fish [8]. These findings support the use of gut microecological analysis to investigate disease susceptibility and progression in cultured fish.

Among numerous aquatic pathogens, *Vibrio harveyi* is one of the important opportunistic pathogens that has received widespread attention in marine aquaculture systems. Recent studies and reviews suggest that *Vibrio harveyi* has a high detection rate and strong pathogenic association across multiple cultured hosts, and is considered an important source of vibriosis [9]. In commercially important marine fish, *Vibrio harveyi* can be associated with high mortality, as well as with impaired intestinal microbial barriers and altered host immune responses, suggesting that the state of the intestinal microbiota may participate in the host’s defense against Vibrio infection [10]. From the perspective of pathogen biology, the pathogenicity of *Vibrio harveyi* is usually considered to be related to multiple virulence-associated factors and differences in genetic background; recent genomic and phenotypic studies have shown that different strains can exhibit divergent virulence profiles, and suggested that mobile genetic elements (such as plasmids) may be involved in the formation of virulence differences [11,12]. Thus, abnormal enrichment or dominance of *Vibrio harveyi* in diseased hybrid porcupinefish should be considered a potential pathogenic signal and interpreted together with host and environmental factors.

Intestinal barrier injury may be a key connecting link between bacterial infection and systemic lesions in fish. The fish intestinal epithelium not only assumes the function of digestion and absorption but also serves as an important mucosal barrier preventing pathogens and their associated products from entering the internal environment. Existing reviews emphasize that there is a close interaction among the intestinal microbiota, barrier integrity, mucosal immunity, and inflammatory responses, and dysbiosis may reduce the host’s ability to resist infection and environmental stress [5,6]. When pathological changes such as intestinal villus atrophy, epithelial cell shedding, lamina propria edema, and inflammatory cell infiltration occur, the intestinal barrier function may decline, providing conditions for the colonization, expansion, or potential translocation of opportunistic pathogens. Although direct evidence for the ‘gut–eye axis’ in fish remains limited, mammalian and ophthalmological studies suggest that the intestinal microbiota can affect ocular health through immune modulation, inflammatory mediators, the blood–retinal barrier, and microbial metabolites [13,14]. For hybrid porcupinefish with intestinal exposure and ocular opacity, the possible links among gut microecological disruption, intestinal barrier injury, and ocular lesions warrant investigation, although any mechanistic interpretation remains hypothetical and requires further validation.

Antimicrobial misuse in aquaculture can facilitate the dissemination of resistant (R) strains and resistance genes; therefore, antimicrobial-resistance risk assessment is an essential component of disease prevention and control. A systematic review and meta-analysis of antimicrobial resistance in bacteria from aquaculture food animals in Asia indicates that resistance has persisted over the past two decades and suggests that aquaculture-associated resistance surveillance is important for both public health and industry security [15]. Furthermore, a review focusing on Southeast Asia highlights the occurrence and transmission risks of resistance in aquaculture systems and proposes strategy directions at the levels of management and monitoring [16]. In addition, a review on Vibrio indicates that Vibrio-associated antimicrobial resistance and pathogenicity in aquaculture environments warrant sustained attention and should be jointly considered when developing control strategies [17]. Therefore, during candidate-pathogen screening and the development of disease-control strategies, investigators should consider both potential shifts in pathogens and antimicrobial-resistance risk to reduce the uncertainty and externality risks associated with empirical antimicrobial use.

Based on the aforementioned background, the clinical manifestations of intestinal exposure and ocular opacity in hybrid porcupinefish may be associated with multiple interacting processes, including disruption of intestinal microecology, impairment of the intestinal barrier, enrichment of opportunistic pathogens, and antimicrobial-resistance risk; however, systematic evidence for these lesions in this hybrid porcupinefish remains limited [1,9]. Therefore, this study aimed to focus on these two typical clinical phenotypes to delineate differential patterns in intestinal microecology and tissue injury, and to evaluate candidate bacterial taxa associated with the lesions as well as potential control clues. By comparing healthy and diseased fish from the same aquaculture setting, this study provides descriptive evidence for etiological assessment of intestinal exposure and ocular opacity and supports subsequent pathogen verification. Integrating clinical signs, gut microbiota, tissue lesions, and antimicrobial-resistance profiles may improve the assessment of disease status in hybrid porcupinefish.

## 2. Materials and Methods

### 2.1. Experimental Fish and Study Design

A case–control design was used. Healthy hybrid porcupinefish served as controls, whereas fish with intestinal exposure or ocular opacity were classified as diseased fish. The experimental fish were hybrid porcupinefish (*Diodon liturosus* ♀ × *Diodon hystrix* ♂) collected in September 2025 from Hainan Sanya Delin Chengxin Aquaculture Company (Sanya, China), with a mean body weight of approximately 320 g and a mean body length of approximately 17 cm.

The experimental fish were categorized into three groups according to clinical signs: a healthy control group, an intestinal exposure group, and an ocular opacity group, with five fish in each group. Fish in the healthy control group showed an intact external appearance without obvious abnormalities such as intestinal exposure, corneal opacity, or skin ulceration; fish in the intestinal exposure group exhibited exposed intestinal or rectal tissues; and fish in the ocular opacity group showed ocular turbidity, corneal opacity, or lens abnormalities. The sample codes were as follows: the healthy control group was coded as CT-CD-JK, the intestinal exposure group as CT-CD-FB, and the ocular opacity group as CT-YQ-FB.

The study compared gut microbial community structure, culturable bacterial composition, histopathological lesions, and antimicrobial susceptibility profiles among the three groups (Table 1). However, because no artificial experimental infection challenge was conducted in this study, the findings were intended to reveal differences in microbiota, pathological changes, and potential pathogen associations between healthy and diseased hybrid porcupinefish, rather than to serve as direct confirmation of causality.

Water-quality and aquaculture-management parameters were recorded during field sampling in September 2025. The three groups were collected from the same indoor cement-pond aquaculture system. The available water-quality records were as follows: water temperature, 27.5–29.2 °C; dissolved oxygen, 5.8–6.7 mg/L; pH, 7.8–8.2; salinity, 28–32 ppt; total ammonia nitrogen, 0.05–0.20 mg/L; nitrite nitrogen, 0.01–0.05 mg/L; and nitrate nitrogen, 1.0–5.0 mg/L. Water temperature was measured using a thermometer, dissolved oxygen using a METTLER TOLEDO DO meter (Mettler-Toledo, Greifensee, Switzerland), and nitrogenous compounds using commercial water-quality test kits from Guangzhou Huankai Microbial Sci. & Tech. Co., Ltd. (Guangzhou, China). Turbidity was not measured and was therefore not included as an estimated water-quality parameter. The aquaculture-management records included a daily water exchange of approximately 80–100 tons, 3–4 water exchanges per day, a stocking density of approximately 16 fish/m^3^, slow pond water flow of approximately 0.1 m/s with an inlet flow of approximately 0.5 m/s, and feeding once daily with Haitong marine fish feed (No. 5).

### 2.2. Clinical Symptom Observation and Sample Collection

Before sampling, the body length, body weight, and main clinical symptoms of each group of hybrid porcupinefish were recorded, and representative photographs of the symptoms were taken. The intestinal exposure group was mainly observed for the degree of intestinal or rectal prolapse, the color of the prolapsed tissue, and surrounding tissue damage; the ocular opacity group was mainly observed for ocular transparency, corneal status, the degree of lens opacity, and periocular tissue changes; and the healthy control group was examined for abnormalities on the body surface, eyes, and abdomen.

The experimental fish were deeply anesthetized with MS-222 before euthanasia and sampling. MS-222 (ethyl 3-aminobenzoate methanesulfonate, i.e., tricaine methanesulfonate) was purchased from Sigma-Aldrich (St. Louis, MO, USA; Cat. No. E10521). The final concentration of the anesthetic solution was 100 mg/L and was buffered to approximately neutral pH with sodium bicarbonate. After deep anesthesia, euthanasia was confirmed by spinal cord transection/pithing before necropsy and sample collection. The fish were then dissected under sterile conditions. Before dissection, the body surface of the fish was rinsed with sterile saline, and the sampling area was surface-disinfected using 75% ethanol.

One intestinal content sample was collected from each fish by gentle scraping with sterile forceps or an inoculation loop. Samples were placed in sterile 50 mL centrifuge tubes or cryovials, labeled, kept on ice, and then stored at −80 °C for gut microbiota sequencing and bacterial isolation. For the ocular opacity group, ocular lesion tissues or eyeball specimens were additionally collected for histopathological observation and pathogen isolation. For the intestinal exposure group and the healthy control group, tissue samples, including the liver, spleen, kidney, foregut, midgut, hindgut, and gallbladder, were collected for histopathological analysis.

To minimize cross-contamination, the investigators collected samples from different individuals and tissues using dedicated sterile instruments or alternatively sterilized the instruments before each sampling.

### 2.3. Extraction of Total DNA from Intestinal Microbiota, 16S rRNA Amplification, and High-Throughput Sequencing

To characterize the gut microbiota, five intestinal content samples from each group were subjected to bacterial 16S rRNA amplicon sequencing. Samples were transported on ice to Shanghai Ling’en Biotechnology Co., Ltd. (Shanghai, China) for microbial DNA extraction, amplicon library preparation, and sequencing.

Total microbial DNA was extracted from intestinal content samples according to the sequencing provider’s standard PacBio 16S rRNA amplicon sequencing workflow. DNA integrity was assessed by 1% agarose gel electrophoresis. To profile bacterial communities, the bacterial 16S rRNA V1-V9 region was amplified using barcode-tagged 27F/1492R primers (27F, 5′-AGAGTTTGATCMTGGCTCAG-3′; 1492R, 5′-TACGGYTACCTTGTTACGACTT-3′).

PCR amplification was performed using TransStart FastPfu DNA Polymerase (TransGen Biotech, Beijing, China; Cat. No. AP221-02) on an ABI GeneAmp 9700 PCR system (Applied Biosystems, Foster City, CA, USA). The 20 μL PCR mixture contained 4 μL of 5× FastPfu Buffer, 2 μL of 2.5 mM dNTPs, 0.8 μL of forward primer (5 μM), 0.8 μL of reverse primer (5 μM), 0.4 μL of FastPfu DNA Polymerase, 10 ng of template DNA, and nuclease-free water to a final volume of 20 μL. The PCR program consisted of initial denaturation at 95 °C for 5 min, followed by 27 amplification cycles of denaturation at 95 °C for 30 s, annealing at 58 °C for 30 s, extension at 72 °C for 45 s, and a final extension at 72 °C for 10 min.

For each sample, PCR amplification was performed in triplicate, and PCR products from the same sample were pooled to reduce amplification bias. The pooled PCR products were checked by 2% agarose gel electrophoresis. Target amplicons were excised from the gel, purified using the AxyPrepDNA Gel Extraction Kit (Axygen Biosciences, Union City, CA, USA), eluted with Tris-HCl buffer, and checked again by 2% agarose gel electrophoresis. The purified amplicons were quantified using the QuantiFluor-ST fluorometric quantification system (Promega Corporation, Madison, WI, USA), and amplicons from different samples were pooled in appropriate proportions according to the required sequencing depth.

PacBio amplicon libraries were constructed from the pooled purified PCR products. Briefly, the amplicons were subjected to end-repair/fill-in processing, ligated with adapters to generate SMRTbell-type library molecules, and treated with exonuclease to remove unligated adapters or incomplete library products. After library quality assessment, sequencing was performed on the PacBio platform (Pacific Biosciences, Menlo Park, CA, USA) by Shanghai Ling’en Biotechnology Co., Ltd. (Shanghai, China) Circular consensus sequence (CCS) reads were generated and exported in FASTQ format for subsequent bioinformatics analysis.

### 2.4. Processing of 16S rRNA Sequencing Data and Bioinformatics Analyses

After quality control, the raw sequencing data were processed to remove adapter sequences, low-quality sequences, short sequences, and chimeric sequences to obtain valid sequences. The valid sequences were then clustered into operational taxonomic units (OTUs) at 97% similarity using pipelines such as QIIME2/VSEARCH, and these OTUs were subsequently used for taxonomic annotation, α-diversity, β-diversity, and community composition analyses.

The sequencing workflow report indicated that PacBio CCS reads were generated and saved in FASTQ format using SMRT Link software (version 9.0; Pacific Biosciences, Menlo Park, CA, USA). Per-sample sequence counts reported in Data.stat.xls were cross-checked against the corresponding .fq.gz files, and FASTQ-level quality metrics were calculated directly from the raw files. The generated QC table included per-sample read counts, total bases, read-length statistics, GC content, N-base proportion, Q20, Q30, and mean Phred quality.

Taxonomic annotation was performed by alignment against the SILVA 138 database to obtain the taxonomic composition of each sample at the phylum, class, order, family, genus, and species levels. To illustrate structural differences in microbial communities across different sample groups, the relative abundances at each taxonomic rank were calculated, and stacked bar plots, heatmaps, or other community composition plots were generated.

α-diversity analysis was used to evaluate the richness and diversity of the intestinal microbial community in each group of samples, primarily including indices such as Observed species, Chao1, Shannon, and Simpson. Meanwhile, rarefaction curves and rank–abundance curves were plotted to assess sequencing depth and community evenness. β-diversity analysis used Bray–Curtis distances to evaluate differences in community structure among different samples, which were visualized using principal coordinates analysis (PCoA) and non-metric multidimensional scaling (NMDS). Differences in community structure among groups were statistically analyzed using the permutational multivariate analysis of variance (PERMANOVA) test with 999 permutations.

Differential taxa were identified using linear discriminant analysis effect size (LEfSe) analysis. Specifically, taxa showing significant between-group differences were first screened using the Kruskal–Wallis rank-sum test, and their effect sizes were then estimated by linear discriminant analysis (LDA). Differential taxa were defined using thresholds of LDA score > 3.0 and *p* < 0.05.

### 2.5. Bacterial Isolation and Purification

To characterize the cultivable bacterial composition in healthy and diseased porcupinefish, samples from the healthy control group, the intestinal exposure group, and the ocular opacity group were collected for bacterial isolation and culture. Specifically, intestinal contents were aseptically sampled, a small amount of each sample was transferred with an inoculating loop, and three-zone streaking was performed on 2216E agar plates. In addition, ocular lesion tissue or ocular exudates from the ocular opacity group were inoculated onto 2216E agar plates using the same procedure.

To support the culture and enumeration of marine bacteria, 2216E agar plates were prepared using 2216E Agar (Qingdao Haibo Biotechnology Co., Ltd. (Qingdao, China), catalog no. HB0132, 250 g).

To obtain purified isolates, inoculated plates were incubated at 30 °C for 18–24 h in a constant-temperature incubator. After incubation, single dominant colonies were selected based on colony morphology, color, size, margin characteristics, and transparency, and were repeatedly streaked onto fresh 2216E agar plates until morphologically uniform single colonies were obtained. Subsequently, purified isolates were cultured in 2216E broth with shaking, mixed with sterile glycerol to a final concentration of 30%, and stored at −80 °C for subsequent analyses.

### 2.6. 16S rRNA-Based Molecular Identification of Isolated Strains

To molecularly identify the purified bacterial isolates, we amplified and sequenced the 16S rRNA gene. Specifically, a single colony was inoculated into 2216E broth and incubated at 30 °C with shaking for 12–18 h. Thereafter, bacterial genomic DNA was extracted from the culture using the TIANamp Bacteria DNA Kit (TIANGEN Biotech, Beijing, China; Cat. No. GDP302-02), which is designed for genomic DNA isolation from both Gram-negative and Gram-positive bacteria; the manufacturer-listed Ref. No. was GDP302-02 (50 preparations).

To obtain near-full-length 16S rRNA sequences, we amplified the 16S rRNA gene using the universal bacterial primers 27F (5′-AGAGTTTGATCMTGGCTCAG-3′) and 1492R (5′-TACGGYTACCTTGTTACGACTT-3′). PCR was conducted with Phusion High-Fidelity PCR Master Mix with HF Buffer (New England Biolabs, Ipswich, MA, USA; Cat. No. M0531S). The 25 μL reaction contained 12.5 μL 2× Phusion High-Fidelity PCR Master Mix, 0.5 μL of primer 27F (10 μmol/L), 0.5 μL of primer 1492R (10 μmol/L), and 1 μL template DNA, with nuclease-free water added to 25 μL. The cycling conditions were as follows: initial denaturation at 98 °C for 30 s; 30 cycles of denaturation at 98 °C for 10 s, annealing at 55 °C for 30 s, and extension at 72 °C for 60 s; and a final extension at 72 °C for 5 min.

To assign taxonomic identities to the isolates, PCR amplicons were verified by 1% agarose gel electrophoresis and then submitted to BGI Genomics (Guangzhou, China) for Sanger sequencing. After sequence assembly and quality trimming, homologous sequences were searched against the NCBI BLAST (BLAST+ version 2.17.0; National Center for Biotechnology Information, Bethesda, MD, USA) database, and the taxonomic placement was inferred based on the closest reference sequence. In general, a 16S rRNA sequence similarity of ≥99% was considered suggestive of species-level assignment, whereas lower similarity or high similarity among closely related species supported only genus-level assignment. However, for taxa with many closely related species (e.g., Vibrio), species-level identification based on a single 16S rRNA locus has limited resolution and should therefore be interpreted cautiously. If needed, additional confirmation may be obtained by analyzing housekeeping genes (e.g., *gyrB*, *rpoD*, and *toxR*) or by whole-genome sequencing.

### 2.7. Antimicrobial Susceptibility Test

Antimicrobial susceptibility of the isolated strains was assessed using the Kirby–Bauer disk diffusion method. The tested agents comprised 30 antibiotics, including cefoperazone (CFP), carbenicillin (CB), penicillin G (P), and furazolidone (FZ); detailed information on drug names, abbreviations, disk contents, and manufacturers is provided in Table S1. Before testing, the isolates stored at −80 °C were streaked onto 2216E agar plates and incubated at 30 °C for 12–18 h for recovery. A single colony was then inoculated into 2216E broth and cultured at 30 °C with shaking until the logarithmic phase. Subsequently, the bacterial suspension was adjusted with sterile normal saline to a 0.5 McFarland standard, corresponding to approximately 1 × 10^8^ colony-forming units (CFU)/mL.

To ensure standardized inoculation for antimicrobial susceptibility testing, 200 μL of the bacterial suspension was evenly spread onto the surface of 2216E agar plates. After the inoculum was fully absorbed, antibiotic disks were placed on the agar surface with sterile forceps and gently pressed to ensure complete contact with the medium. The plates were subsequently incubated at 30 °C for 18–24 h. After incubation, inhibition zone diameters around each disk were measured using a vernier caliper and recorded in mm. For each strain–antibiotic combination, three technical replicates were performed, and the mean value was used for subsequent analyses.

Interpretation of antimicrobial susceptibility results was performed according to the Clinical and Laboratory Standards Institute (CLSI) guidance for bacteria from aquatic animals where applicable, including VET03 and VET04. CLSI VET03 provides procedures for broth microdilution and disk diffusion testing of bacteria of aquatic-animal origin, whereas CLSI VET04 provides interpretive criteria based on VET03, including drug selection, quality control, clinical breakpoints, and epidemiological cutoff values. Based on inhibition zone diameters, isolates were categorized as susceptible (S), intermediate (I), or resistant (R) when applicable aquatic-animal interpretive criteria were available. For isolate–antimicrobial combinations for which aquatic-animal-specific breakpoints were unavailable or not fully validated, the results were interpreted cautiously and summarized mainly as inhibition-zone profiles or tentative R/I/S categories rather than definitive clinical susceptibility classifications.

### 2.8. Histopathology

To characterize tissue-level differences between healthy and diseased porcupinefish, samples from a healthy control group, an intestinal exposure group, and an ocular opacity group were collected for histopathological analysis. In the healthy control and intestinal exposure groups, the liver, spleen, kidney, foregut, midgut, hindgut, and gallbladder were sampled; in the ocular opacity group, the eyeball, periocular lesions, and intestinal tissues were sampled. All tissues were immediately immersed in 4% paraformaldehyde fixative upon collection and fixed at 4 °C for 24 h. The 4% paraformaldehyde fixative used was Paraformaldehyde Fixative (Neutral) (Servicebio, Wuhan, China, Cat. No. G1101-500ML).

The fixed tissues were rinsed under running water and then processed for routine paraffin sectioning. Tissue samples were sequentially dehydrated through graded ethanol (75%, 85%, 95%, and absolute ethanol), cleared in xylene, infiltrated with paraffin, and embedded. After trimming, the paraffin blocks were sectioned into serial 5 μm-thick slices, flattened in 40 °C warm water, and mounted onto polylysine-coated slides. The sections were baked at 60 °C for 2 h and subsequently subjected to hematoxylin and eosin (H&E) staining.

H&E staining was performed using an H&E Staining Kit (Servicebio, Wuhan, China, Cat. No. G1005-500ML). The sections were deparaffinized in xylene, rehydrated through graded ethanol, stained with hematoxylin, differentiated, blued, counterstained with eosin, dehydrated through graded ethanol, cleared in xylene, and mounted with neutral resin, after which they were observed and photographed under a light microscope.

For intestinal tissues, the observation focused on villus integrity, mucosal epithelial cell arrangement, goblet cell changes, lamina propria edema, inflammatory cell infiltration, and intestinal wall structural integrity. For ocular tissues, the observation focused on structural changes in the cornea, lens, iris, retina, and eyeball wall. For the liver, spleen, kidney, and gallbladder tissues, pathological changes such as cellular degeneration, necrosis, congestion, hemorrhage, and inflammatory cell infiltration were mainly observed.

ImageJ software (version 1.54p; National Institutes of Health, Bethesda, MD, USA) was used for the quantitative analysis of intestinal histopathological damage. For each fish, five clear fields of view were randomly selected to measure villus height, intestinal wall thickness, and goblet cell number. The average value of the five fields of view for each index was taken as the statistical value of the individual fish, and intergroup comparisons were conducted using individual fish as biological replicates.

### 2.9. Statistical Analysis

All statistical analyses were conducted using R software (version 4.4.2; R Foundation for Statistical Computing, Vienna, Austria). Data are presented as mean ± standard deviation (SD). Before statistical testing, data on microbial α-diversity indices, relative abundances of dominant taxa, quantitative histopathology metrics, and inhibition zone diameters from antimicrobial susceptibility assays were assessed for normality and homogeneity of variance.

For comparisons among three groups, data meeting the assumptions of normality and homogeneity of variance were analyzed using one-way analysis of variance (ANOVA), followed by Tukey’s multiple-comparison test for pairwise contrasts; in contrast, data violating normality or homogeneity of variance were analyzed using the Kruskal–Wallis nonparametric test, followed by Dunn’s test for pairwise comparisons. For comparisons between two groups, normally distributed data were analyzed using Student’s *t*-test, whereas non-normally distributed data were analyzed using the Wilcoxon rank-sum test. Multiple comparisons were controlled using the Benjamini–Hochberg method for false discovery rate (FDR) correction. A *p*-value < 0.05 was considered statistically significant, and a *p*-value < 0.01 was considered highly significant.

Between-group differences in β-diversity were evaluated using PERMANOVA with 999 permutations. In LEfSe analysis, differential taxa were screened using thresholds of *p* < 0.05 and LDA score > 3.0. Bacterial isolation and identification outcomes were summarized by the composition and detection proportions of isolates in each group, and differences in the detection rates of target strains among groups were assessed using Fisher’s exact test. Antimicrobial susceptibility results were summarized using inhibition zone diameters, susceptibility categories, R/I/S category proportions, and R-category proportions where appropriate.

### 2.10. Animal Ethics Statement

This study used samples obtained from naturally diseased and healthy farmed hybrid porcupinefish. All sampling and experimental procedures complied with the requirements for aquatic animal research ethics and animal welfare of Hainan Tropical Ocean University and were approved by the Institutional Animal Care and Use Committee of Hainan Tropical Ocean University (approval no. 2026-KJCLL-048). Before necropsy and sampling, experimental fish were deeply anesthetized with 100 mg/L MS-222 buffered to approximately neutral pH, and euthanasia was confirmed by spinal cord transection/pithing before tissue collection.

## 3. Results

### 3.1. Clinical Symptoms and Histopathological Changes

**Figure 1:**
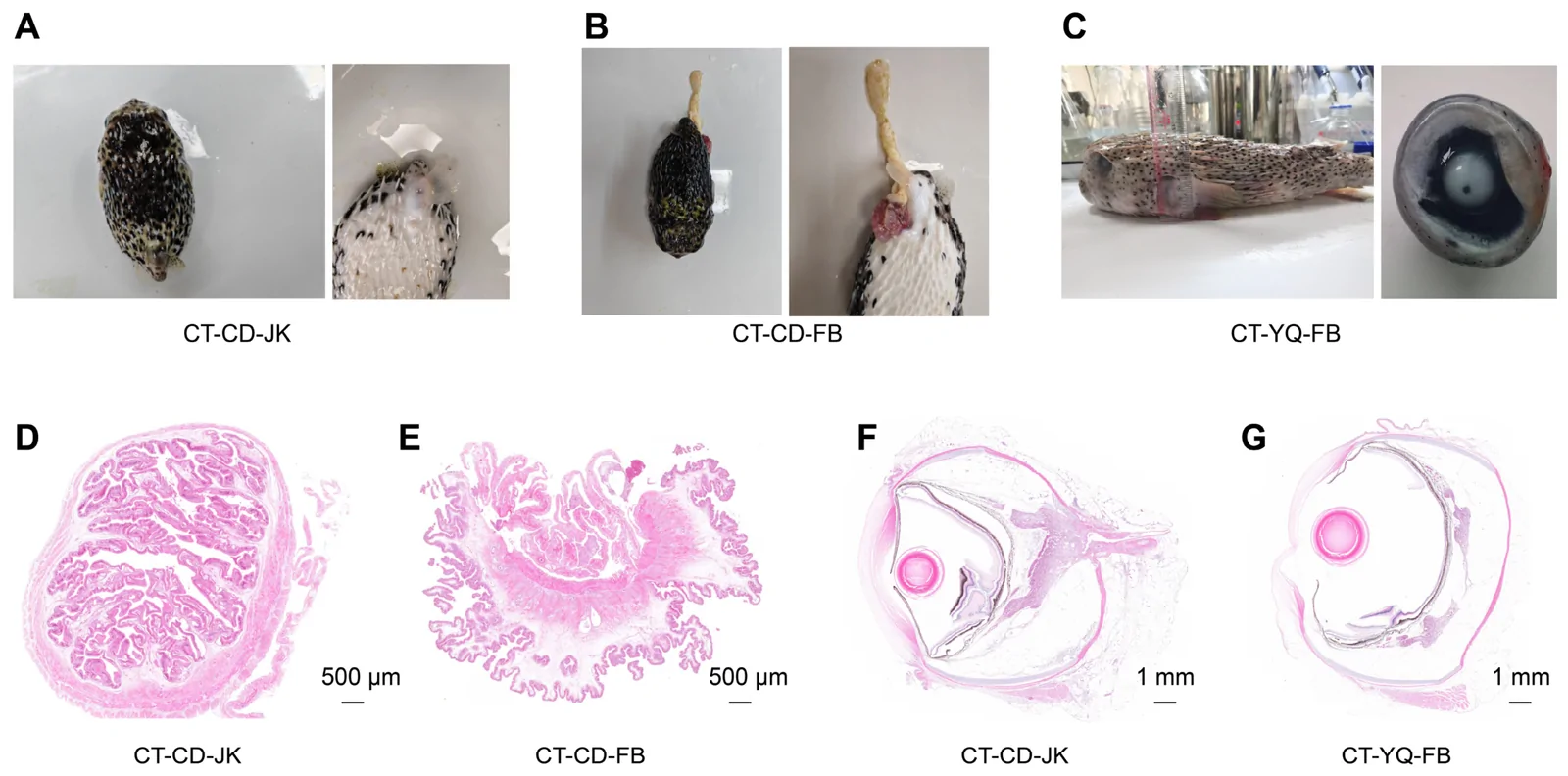
Clinical symptoms and histopathological changes. (**A**) Representative gross appearance of the healthy control group (CT-CD-JK); (**B**) Representative gross appearance of the intestinal exposure group (CT-CD-FB), showing intestinal exposure; (**C**) Representative gross appearance and isolated eyeball of the ocular opacity group (CT-YQ-FB), showing corneal opacity; (**D**) H&E-stained midgut section of the healthy control group; (**E**) H&E-stained midgut section of the intestinal exposure group; (**F**) H&E-stained eyeball section of the healthy control group; (**G**) H&E-stained eyeball section of the ocular opacity group. Scale bars: midgut sections (**D**,**E**) = 500 μm; eyeball sections (**F**,**G**) = 1 mm.

Clinical phenotypes differed among the three groups of hybrid porcupinefish. The CT-CD-JK group appeared normal in appearance, with no observed intestinal exposure, corneal opacity, or overt body surface injury; the CT-CD-FB group exhibited exposed intestinal or rectal tissue, with localized swelling and hyperemia of the exposed tissue; the CT-YQ-FB group showed corneal opacity, decreased ocular transparency, and abnormal periocular tissues (Figure 1A–C).

H&E staining results showed that the midgut tissue structure of the CT-CD-JK group was intact, the intestinal wall outline was clear, and the intestinal villi were neatly arranged and continuous in structure; the midgut tissue of the diseased fish showed obvious pathological damage, characterized by an irregular intestinal outline, structural destruction of the intestinal wall, villus atrophy or fusion, epithelial cell shedding, lamina propria edema, and inflammatory cell infiltration (Figure 1D,E). Quantitative histopathological analysis of the midgut showed that the intestinal injury-related indices of the diseased groups were significantly altered compared to the healthy control group, with the most obvious damage observed in the CT-CD-FB group (Table 2). Observation of ocular tissue showed that the eyeball wall of the CT-CD-JK group had clear layers and an intact lens structure; in the CT-YQ-FB group, the eyeball wall was swollen with uneven thickness, local tissue layers were blurred, and the periocular soft tissues showed edematous and loose changes (Figure 1F,G).

### 3.2. Sequencing Depth and α-Diversity of the Intestinal Microbiota

**Figure 2:**
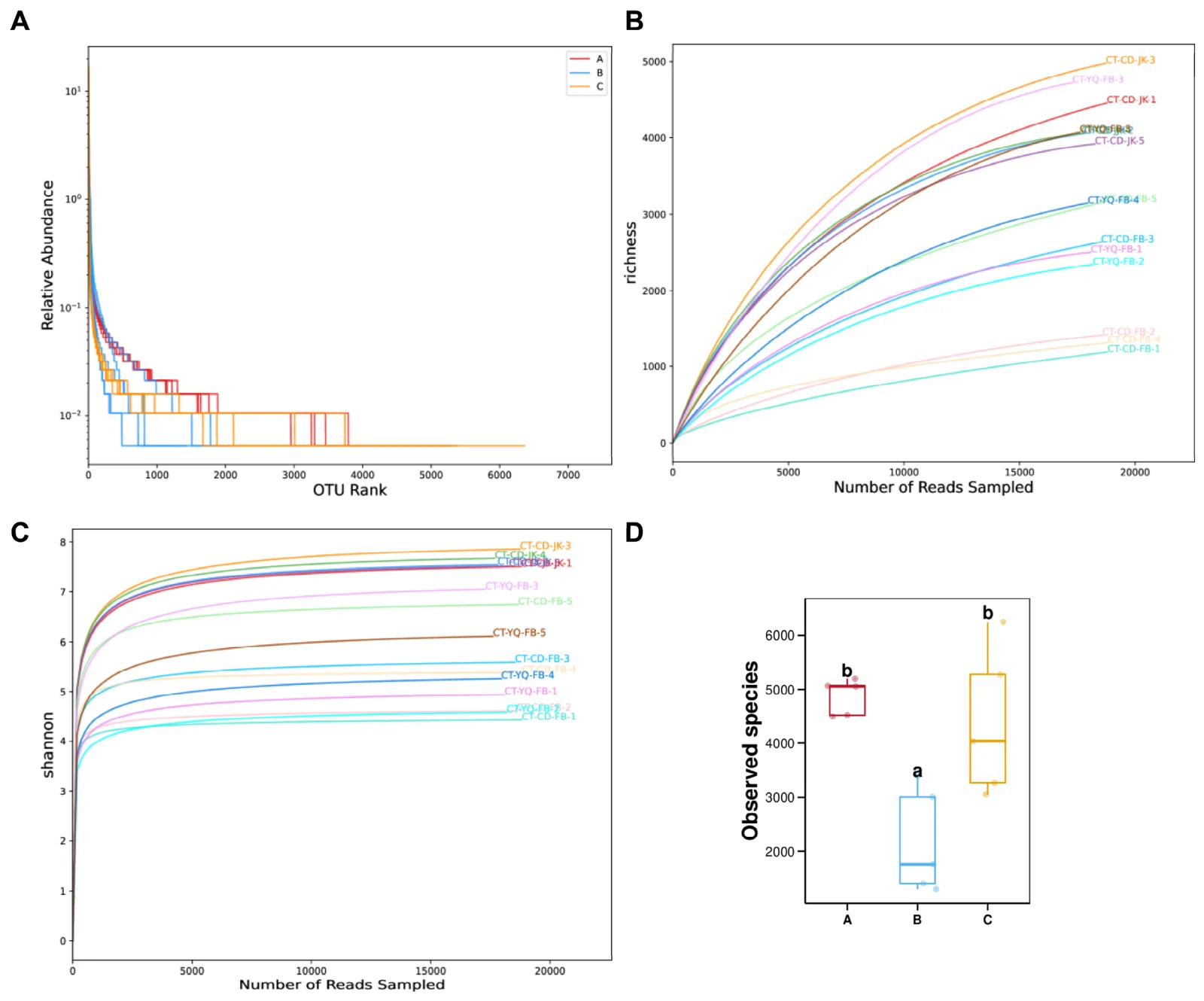
Gut microbiota sequencing depth and alpha-diversity analysis. (**A**) OTU rank-abundance curves of the three sample groups, reflecting microbial richness and evenness. In the legend and *x*-axis labels, A, B, and C represent the CT-CD-JK, CT-CD-FB, and CT-YQ-FB groups, respectively. (**B**) Species-richness rarefaction curves for individual samples, showing cumulative richness trends as sequencing reads increase. (**C**) Shannon diversity rarefaction curves for individual samples, showing diversity trends as sequencing reads increase. The upward trends in panels B and C reflect within-sample accumulation with increasing sequencing depth and do not indicate an intergroup increase. (**D**) Boxplots of the observed species index for the three sample groups; A, B, and C on the *x*-axis represent CT-CD-JK, CT-CD-FB, and CT-YQ-FB, respectively. Different lowercase letters indicate significant differences between groups (*p* < 0.05), whereas identical letters indicate no significant difference (*p* > 0.05). CT-CD-JK: healthy control; CT-CD-FB: intestinal exposure; CT-YQ-FB: ocular opacity; n = 5 per group.

Sequencing depth and gut microbiota diversity were assessed using OTU rank–abundance curves, rarefaction curves, and alpha-diversity indices in the CT-CD-JK, CT-CD-FB, and CT-YQ-FB groups. The OTU rank–abundance curves showed that all three groups exhibited a typical long-tail distribution, indicating that a small number of high-abundance OTUs dominated the community, whereas a large number of low-abundance OTUs constituted the rare microbiota (Figure 2A). The species-richness and Shannon rarefaction curves described cumulative richness and diversity trends within individual samples as sequencing reads increased; these curves gradually approached a plateau, supporting the adequacy of sequencing depth for downstream community-structure analysis (Figure 2B,C). Across the 15 PacBio 16S rRNA amplicon sequencing libraries, 506,417 CCS reads were obtained in total, with 30,181–40,106 CCS reads per sample. The per-sample read counts calculated directly from the .fq.gz FASTQ files were identical to the sequence counts reported in the sequencing summary file. FASTQ-derived QC metrics showed mean read lengths of approximately 1.44–1.45 kb, Q20 values of 98.60–98.93%, Q30 values of 96.84–97.58%, GC contents of 54.00–55.32%, and no detected N bases. Detailed per-sample reads and QC metrics are provided in Table S2, and the corresponding NCBI BioProject and SRA accession information for these 15 samples is provided in Table S3.

The observed species index results indicated differences in species richness among the three sample groups. In Figure 2D, A, B, and C represent the CT-CD-JK, CT-CD-FB, and CT-YQ-FB groups, respectively. The number of observed species in the CT-CD-JK and CT-YQ-FB groups was higher than that in the CT-CD-FB group. Specifically, the median was approximately 5000 in the CT-CD-JK group, 4100 in the CT-YQ-FB group, and 1700 in the CT-CD-FB group, suggesting reduced gut microbiota richness in the intestinal exposure group (Figure 2D). Other alpha-diversity indices, including Chao1, Shannon, and Simpson, were also evaluated as part of the alpha-diversity analysis. Additional observed-species violin plots and OTU accumulation analyses are provided in Figure S1A,B, respectively, whereas shared/core microbiota analyses are shown in Figure S2A–D.

### 3.3. β Diversity and Differences in the Overall Structure of Intestinal Microbiota

**Figure 3:**
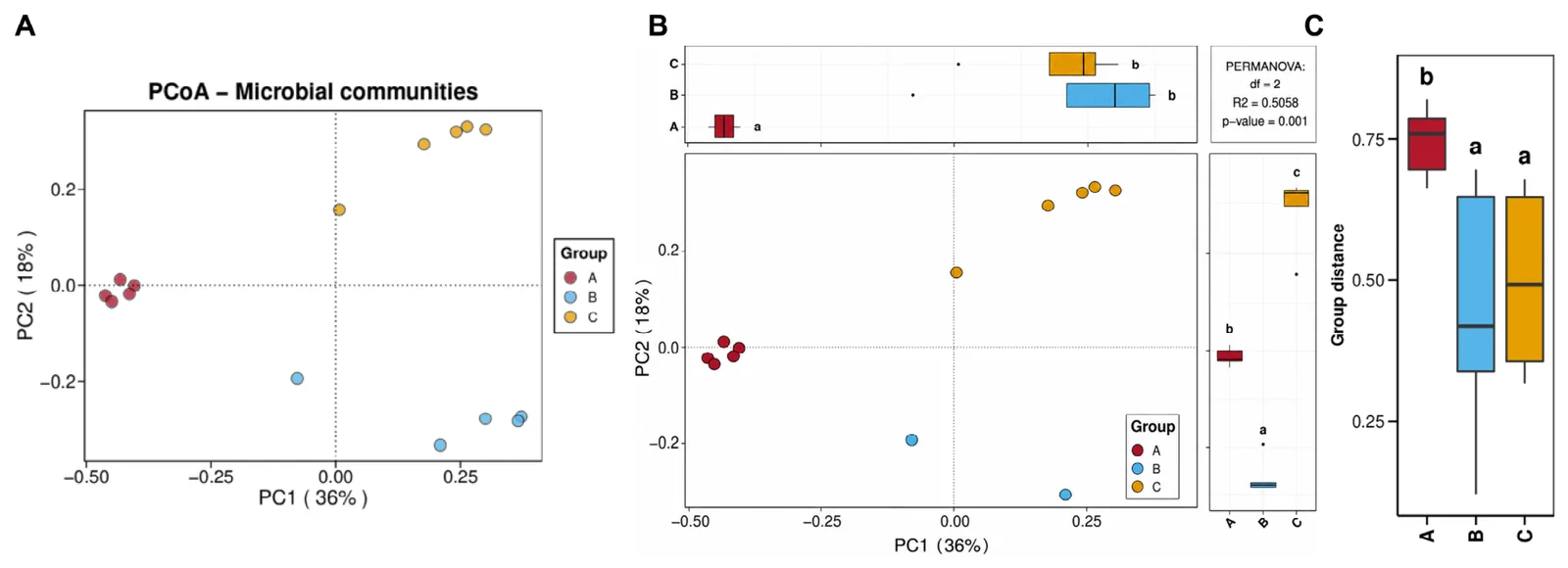
β-diversity analysis of the gut microbiota in three groups of samples. (**A**) PCoA based on the community distance matrix; (**B**) PCoA ordination with marginal distributions and PERMANOVA summary for intergroup differences; (**C**) boxplot of intergroup distances among the three groups. In the figure, A, B, and C represent the CT-CD-JK, CT-CD-FB, and CT-YQ-FB groups, respectively. CT-CD-JK: healthy control group; CT-CD-FB: intestinal exposure group; CT-YQ-FB: ocular opacity group. PERMANOVA: df = 2, R^2^ = 0.5058, *p* = 0.001. Different letters indicate significant differences between groups, *p* < 0.05.

β-diversity was assessed using PCoA and between-group distance analysis to compare gut microbiota structure among the three groups. The PCoA results indicated that the CT-CD-JK, CT-CD-FB, and CT-YQ-FB groups were distinctly separated in ordination space, with strong within-group clustering, suggesting marked differences in porcupinefish intestinal microbiota structure across clinical conditions (Figure 3A). The first two principal coordinates explained 54% of the community variation, with PC1 accounting for 36% and PC2 for 18% (Figure 3A).

PERMANOVA showed that the grouping factor had a significant impact on the intestinal microbiota structure (df = 2, R^2^ = 0.5058, *p* = 0.001), indicating that the grouping factor could explain approximately 50.58% of the community variation (Figure 3B; Table 3). In Figure 3B, the PCoA ordination is presented together with marginal distributions and a PERMANOVA summary to clarify the grouping effect. The between-group distance analysis showed a larger microbiota structure distance between the CT-CD-JK group and the diseased groups, whereas the distance between CT-CD-FB and CT-YQ-FB was relatively close (Figure 3C). Supplementary PCA and NMDS ordinations further supported within-group clustering and between-group separation (Figure S3A,B).

The inter-sample similarity heatmap and unweighted pair group method with arithmetic mean (UPGMA) clustering tree further supported that the three groups of samples possessed a distinct pattern of within-group clustering and between-group separation (Figure S3C–E).

### 3.4. Differences in Species Composition of Intestinal Microbiota

**Figure 4:**
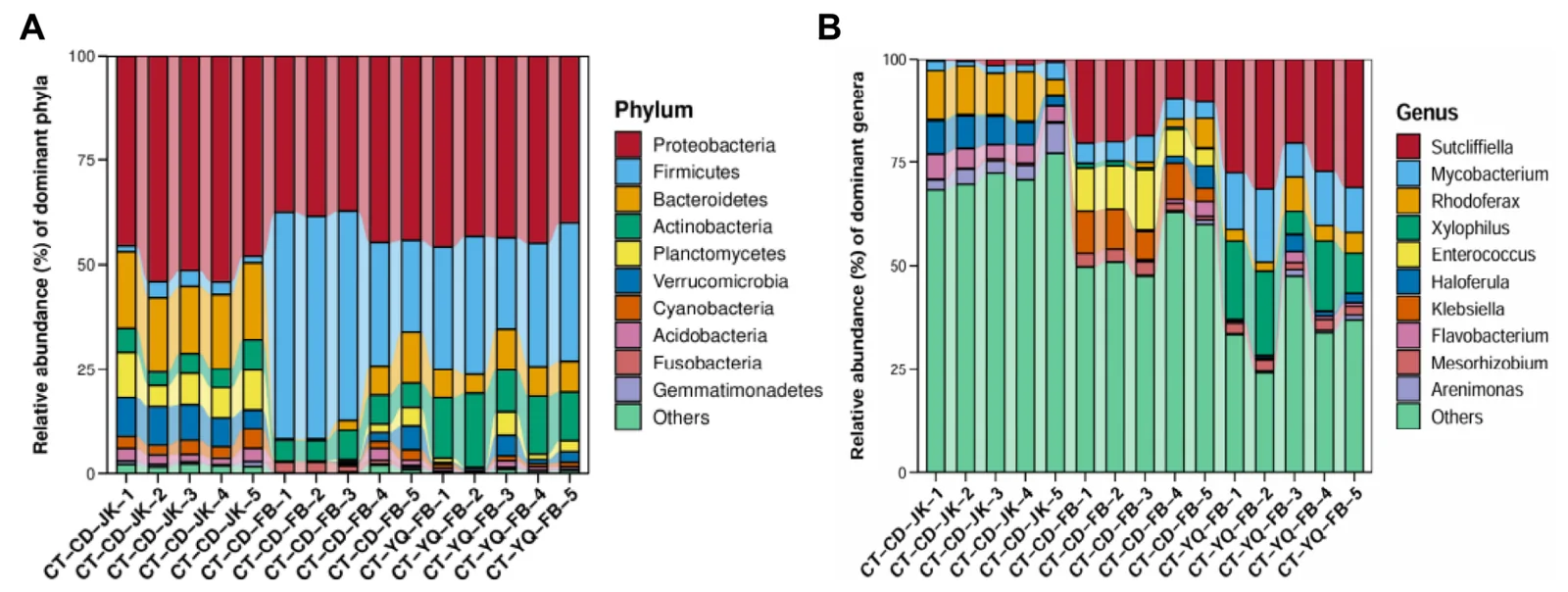
Phylum- and genus-level composition of the gut microbiota in the three groups. (**A**) Stacked bar plot of the relative abundance of dominant taxa at the phylum level; (**B**) Stacked bar plot of the relative abundance of dominant taxa at the genus level. Only major taxa with relatively high abundance are shown; low-abundance taxa are merged into “Others”. CT-CD-JK: healthy control group; CT-CD-FB: intestinal exposure group; CT-YQ-FB: ocular opacity group. n = 5 per group.

The intestinal microbiota of the three groups of porcupinefish showed obvious compositional differences at both the phylum and genus levels. Phylum-level analysis revealed that Proteobacteria was the common dominant phylum across all three groups, with the CT-CD-JK group showing a higher relative abundance of Proteobacteria, at approximately 50–75%. In the CT-CD-FB group, Firmicutes increased significantly and became the main dominant phylum, at approximately 60–75%. In the CT-YQ-FB group, the distribution of phyla, including Proteobacteria, Firmicutes, Bacteroidetes, and Actinobacteria, was relatively balanced (Figure 4A).

Genus-level profiling indicated that the dominant genera differed across the three groups. Specifically, *Sutcliffiella* exhibited a higher relative abundance in the CT-YQ-FB group; in contrast, *Rhodoferax* and *Mycobacterium* were the predominant taxa in the CT-CD-JK group. Moreover, the genus-level composition of the CT-CD-FB group was intermediate between the other two groups, with taxa such as *Mycobacterium*, *Haloferula*, *Enterococcus*, and *Xylophilus* also detected (Figure 4B). These findings indicate that both intestinal exposure and ocular opacity were associated with changes in gut microbiota composition.

As supplementary results, compositional profiles at the class, order, family, and species levels are shown in Figure S4A–D, and dominant-phylum distribution patterns are summarized in Figure S4E–H.

### 3.5. LEfSe Differential Microbiota Analysis

**Figure 5:**
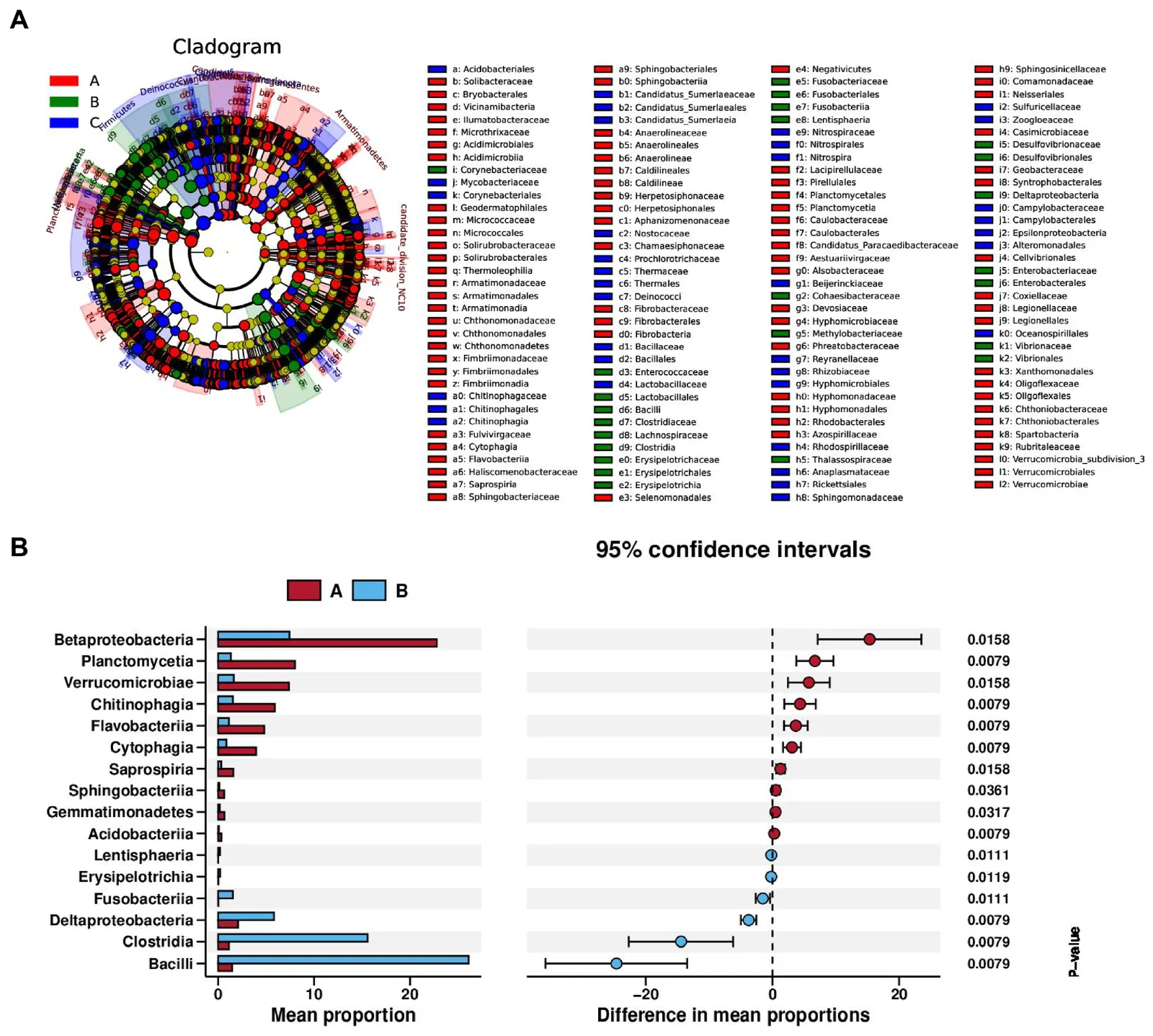
Differential microbial taxa among the three groups of samples. (**A**) LEfSe cladogram showing the phylogenetic distribution of differential microbial taxa among the three groups; (**B**) between-group differential abundance plot showing taxa with significant differences in mean proportions between the CT-CD-JK and CT-CD-FB groups, with 95% confidence intervals. Differential taxa were screened using the criteria LDA score > 3.0 and *p* < 0.05.

LEfSe analysis further identified differentially enriched taxa among the three groups. The CT-CD-JK group was mainly enriched in Sphingobacteriaceae, Cytophagaceae, and Haliscomenobacteraceae, which were largely assigned to Bacteroidetes-related lineages; however, the CT-CD-FB group was enriched in Fusobacteriaceae, Clostridiaceae, and Lactobacillaceae, which were primarily distributed within Firmicutes- and Fusobacteria-related lineages. In addition, the CT-YQ-FB group was enriched in Campylobacteraceae, Oceanospirillaceae, and Vibrionaceae, which were mainly concentrated in Proteobacteria-related lineages (Figure 5A; Table S4). The between-group differential abundance plot further showed taxa with significant differences in mean proportions between the CT-CD-JK and CT-CD-FB groups (Figure 5B).

### 3.6. Isolation of Culturable Strains and 16S rRNA Molecular Identification

This study isolated a total of 23 cultivable bacterial strains from intestinal contents collected from the CT-CD-FB and CT-CD-JK groups, including 11 strains from the CT-CD-FB group and 12 from the CT-CD-JK group. Based on 16S rRNA sequence alignment, the isolates predominantly belonged to the genus *Vibrio* (Table 4; Table S5).

**Figure 6:**
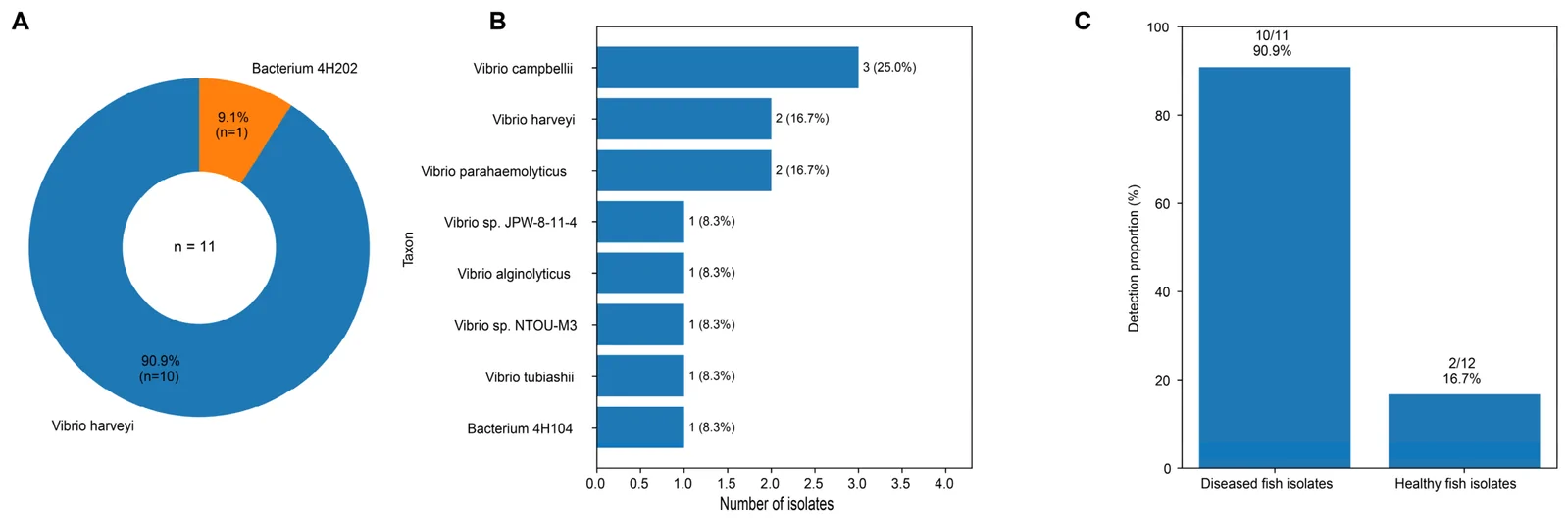
Composition of culturable isolates and detection rate of *Vibrio harveyi*. (**A**) Species composition of isolates obtained from intestinal contents in the CT-CD-FB group; (**B**) Species composition of isolates obtained from intestinal contents in the CT-CD-JK group; (**C**) Detection rate of *Vibrio harveyi* among isolates obtained from intestinal contents in the CT-CD-FB and CT-CD-JK groups. A total of 11 isolates were obtained from intestinal contents in the CT-CD-FB group, and 12 isolates were obtained from intestinal contents in the CT-CD-JK group.

The isolate composition differed between groups. In the CT-CD-FB group, isolates assigned as putative *Vibrio harveyi* based on 16S rRNA sequence alignment accounted for 90.9% of isolates (10 isolates); in addition, one isolate was identified as Bacterium 4H202 (Figure 6A). In contrast, 12 isolates were detected in the CT-CD-JK group, including *Vibrio harveyi*, *Vibrio campbellii*, *Vibrio tubiashii*, *Vibrio alginolyticus*, *Vibrio parahaemolyticus*, and an unnamed *Vibrio* sp. (Figure 6B); among these, *Vibrio harveyi* accounted for 2 isolates (16.7%) (Figure 6C; Table 4).

These results indicate that the proportion of isolates assigned as putative *Vibrio harveyi* by 16S rRNA sequencing was higher in the CT-CD-FB group than in the CT-CD-JK group. Therefore, the higher detection proportion of putative *Vibrio harveyi* may be associated with intestinal microecology dysbiosis and intestinal lesions in porcupinefish with intestinal exposure, although species-level assignment and pathogenicity require further confirmation.

### 3.7. Antimicrobial Susceptibility Analysis of the Isolates

**Figure 7:**
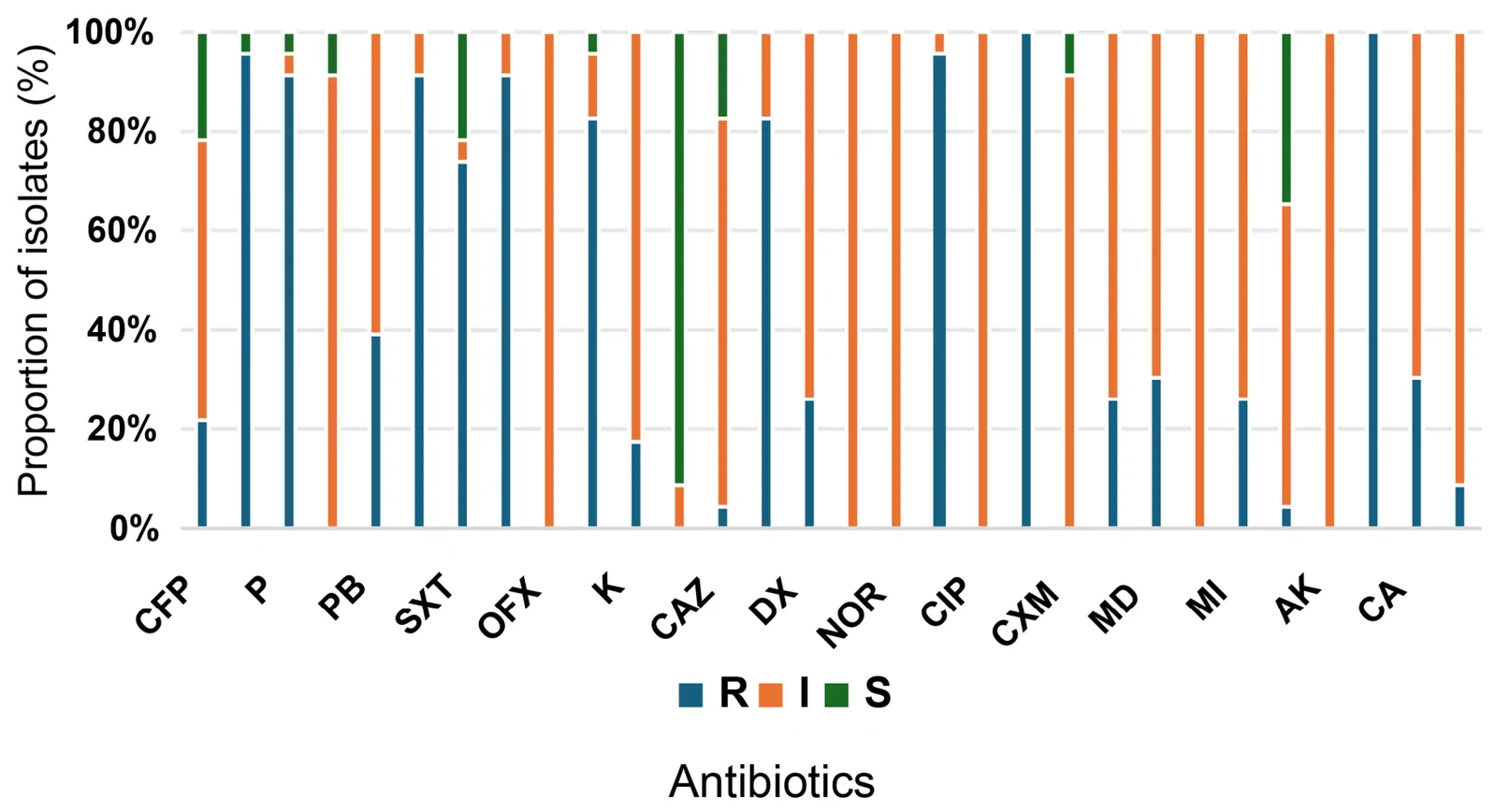
Antimicrobial susceptibility profiles of 23 isolated strains to 30 antibiotics. The figure shows a stacked bar chart of the proportions of isolates categorized as resistant (R), intermediate (I), or susceptible (S) for each of the 30 antibiotics. The *y*-axis indicates the proportion of isolates (%). R, resistant; I, intermediate; S, susceptible. CFP, cefoperazone; P, penicillin G; PB, polymyxin B; SXT, sulfamethoxazole-trimethoprim; OFX, ofloxacin; K, kanamycin; CAZ, ceftazidime; DX, doxycycline; NOR, norfloxacin; CIP, ciprofloxacin; CXM, cefuroxime; MD, midecamycin; MI, minocycline; AK, amikacin; CA, cephalexin.

In this section, antimicrobial susceptibility of the 23 isolates was assessed against 30 antibiotics using the Kirby–Bauer disk diffusion method. The results indicate marked inter-strain variability in susceptibility profiles across antibiotic classes; moreover, the overall pattern suggested multidrug resistance (Figure 7; Table 5).

The R/I/S category distribution showed that a large proportion of isolates was categorized as resistant (R) to multiple beta-lactams and several commonly used antimicrobials. Specifically, the R-category proportion exceeded 80% for CB, P, E, ampicillin (AM), piperacillin (PIP), tetracycline (TE), clindamycin (CC), oxacillin (OX), and vancomycin (VA), whereas the corresponding R-category proportion for sulfamethoxazole-trimethoprim (SXT) was 74% (Figure 7; Table 5).

In contrast, several antibiotics showed a higher proportion of susceptible (S) isolates in vitro. Ceftriaxone (CTR) showed the highest S-category proportion (91%), and the R-category proportions for FZ, ofloxacin (OFX), kanamycin (K), ceftazidime (CAZ), gentamicin (GM), norfloxacin (NOR), ciprofloxacin (CIP), cefuroxime (CXM), cefazolin (CZ), chloramphenicol (C), amikacin (AK), and neomycin (N) were all below 20% (Figure 7; Table 5).

Overall, the 23 isolates showed marked heterogeneity in antimicrobial susceptibility profiles, with larger R-category proportions for selected beta-lactams and several other antimicrobials and larger S-category proportions for selected cephalosporins, quinolones, aminoglycosides, and chloramphenicol. Therefore, these findings suggest a potential risk of multidrug resistance, and subsequent antimicrobial selection should be guided by isolate-specific antimicrobial susceptibility testing.

## 4. Discussion

This study examined two lesions in hybrid porcupinefish—intestinal exposure and ocular opacity—and characterized gut microbiota structure, histopathology, culturable bacteria, and antimicrobial susceptibility profiles. The intestinal exposure group, ocular opacity group, and healthy control group differed substantially in clinical phenotypes, gut microbiota architecture, and tissue injury features. Compared with the healthy controls, the intestinal exposure group showed a reduced observed species index, suggesting decreased gut microbiota richness. Furthermore, PCoA and PERMANOVA analyses indicated significant separation in overall community structure among the three groups, suggesting pronounced remodeling of the intestinal microbiome across distinct clinical states. Fish gut microbiota are considered closely associated with nutrient absorption, epithelial renewal, immune maturation, disease resistance, and environmental adaptation, and dysbiosis is often linked to unfavorable health outcomes and disrupted immune homeostasis [4,6]. Consistently, prior systematic reviews have noted that host species, diet, developmental stage, salinity, water quality, and pathogen exposure can all influence gut microbiota composition, and that disease or stress more frequently coincides with reshaping of dominant taxa and reduced community stability [6]. The reduced richness in the intestinal exposure group is consistent with reports of altered gut microbiota under disease or stress conditions. Meanwhile, the ocular opacity group also displayed a community composition distinct from that of the healthy group, suggesting that individuals with ocular lesions may not be limited to localized ocular abnormalities but may also exhibit altered intestinal microecology. Notably, this study was a case–control observational investigation and therefore cannot establish gut dysbiosis as the initiating cause of intestinal exposure or corneal opacity. Nevertheless, by integrating evidence from gut microbiota, pathological injury, and culturable pathogenic bacteria, this study provides a relatively comprehensive descriptive dataset for diseases affecting hybrid porcupinefish and establishes a foundation for subsequent mechanistic validation.

Environmental factors are important potential contributors to fish disease and microbiome variation. Previous studies have shown that water temperature, dissolved oxygen, salinity, nitrogenous compounds, stocking density, and feeding or water-exchange management can influence fish gut microbial communities and disease susceptibility, and environmental stress may interact with opportunistic pathogens in aquaculture systems [3,4,5,6]. In the present study, the three groups were collected from the same indoor cement-pond aquaculture system, and available field records showed water temperature of 27.5–29.2 °C, dissolved oxygen of 5.8–6.7 mg/L, pH of 7.8–8.2, salinity of 28–32 ppt, total ammonia nitrogen of 0.05–0.20 mg/L, nitrite nitrogen of 0.01–0.05 mg/L, and nitrate nitrogen of 1.0–5.0 mg/L. Turbidity was not measured and was therefore not included as an estimated water-quality parameter. The aquaculture-management records included daily water exchange of approximately 80–100 tons, 3–4 water exchanges per day, stocking density of approximately 16 fish/m^3^, slow pond water flow of approximately 0.1 m/s with inlet flow of approximately 0.5 m/s, and feeding once daily with Haitong marine fish feed (No. 5). These records provide environmental and management context for interpreting the sampled fish, and reduce, but do not eliminate, uncertainty related to culture conditions. However, because the present study did not include longitudinal water-quality monitoring, independent control ponds, or experimental manipulation of environmental variables, these data cannot establish whether environmental fluctuations contributed to disease onset. Therefore, environmental stress cannot be completely excluded as a contributor to the observed clinical signs and microbiome changes.

At the compositional level, this study identified pronounced differences among the three groups at the phylum, family, and genus levels. At the phylum level, the healthy control group was dominated by Proteobacteria, whereas Firmicutes increased markedly in the intestinal exposure group and became a major dominant lineage. Recent reviews suggest that Proteobacteria, Firmicutes, Bacteroidetes, and Actinobacteria commonly constitute core components of fish gut microbiota, but their relative abundances vary substantially with host type, aquaculture environment, and health status [4,6]. Therefore, the elevated Firmicutes observed in the intestinal exposure group may reflect disease-associated shifts in intestinal niches, changes in nutrient substrates, or intensified inflammation-related selection pressures. At the genus level, *Sutcliffiella* showed relatively high abundance in the ocular opacity group; Rhodoferax and Mycobacterium mainly characterized the healthy control group; and the intestinal exposure group exhibited a comparatively more even genus-level composition, including *Mycobacterium*, *Haloferula*, *Enterococcus*, and *Xylophilus*. LEfSe analysis further showed that the healthy control group was enriched in Sphingobacteriaceae, Cytophagaceae, and Haliscomenobacteraceae, the intestinal exposure group was enriched in Fusobacteriaceae, Clostridiaceae, and Lactobacillaceae, and the ocular opacity group was enriched in Campylobacteraceae, Oceanospirillaceae, and Vibrionaceae. These results suggest that different lesion types may be associated with distinct gut microbiota profiles. Compared with previous fish disease–microbiome studies, a key feature of this work is the concurrent inclusion of intestinal exposure and corneal opacity phenotypes, enabling results that not only capture differences between health and disease but also provide preliminary evidence for microbiota heterogeneity across lesion types. Systematic reviews likewise indicate that disease-associated dysbiosis in fish does not necessarily manifest as an absolute dominance of a single pathogen, but may instead involve broader shifts across multiple taxa [6]. In the present study, however, microbial co-occurrence or network analysis and functional prediction were not performed. Accordingly, the increased Firmicutes, the enrichment of Vibrionaceae, and the additional shifts in several low-to-medium-abundance taxa observed here should be interpreted as descriptive community-composition patterns rather than direct evidence of microbial interaction networks or functional effects. Future studies integrating ASV-based analysis, functional prediction or metagenomic sequencing, metabolomics, and host transcriptomics may help clarify potential functional changes in the microbiota and their links to inflammation and barrier integrity.

Histopathology indicated that diseased hybrid porcupinefish exhibited marked injuries in both the intestine and the eye. In the intestinal exposure group, the intestine showed disruption of the intestinal wall architecture, villus atrophy or fusion, epithelial cell sloughing, lamina propria edema, and inflammatory cell infiltration. In contrast, the ocular opacity group was characterized by swelling of the eyeball wall, blurred tissue stratification, and edema-like changes in the periocular tissues. Because the fish intestine functions not only in digestion and absorption but also as a critical mucosal immune barrier, compromised epithelial integrity may facilitate contact between opportunistic pathogens (and their associated factors) and the mucosal lamina propria, thereby triggering localized inflammation. A review on Vibrio infections has noted that vibriosis can present with diverse clinical manifestations, including skin lesions, hemorrhage, septicemia, gastroenteritis, visceral lesions, and ocular lesions [1]. Moreover, studies and reviews focusing on *Vibrio harveyi* have emphasized that this bacterium is closely associated with vibriosis events in mariculture systems and may co-occur with multiple tissue-injury phenotypes [1,9]. Therefore, the intestinal tissue injuries observed in this study are broadly consistent with the pathological features reported for Vibrio infections, particularly mucosal disruption and inflammatory cell infiltration, suggesting that the intestinal barrier in diseased fish may be compromised. Similarly, the disorganization of ocular tissue structure in the ocular opacity group resembles the ocular injuries commonly described in Vibrio infections. However, the present study does not provide direct evidence for a gut–eye relationship or demonstrate that ocular lesions resulted from the migration of gut-derived pathogens. Because pathogen isolation from ocular lesions was limited, the ocular pathology observed in this study should not be attributed to a specific gut-derived pathogen. A more cautious interpretation is that ocular opacity was accompanied by ocular histopathological injury and altered intestinal microbiota, but these concurrent findings do not establish a causal gut–eye mechanism. Future work should include targeted pathogen isolation from ocular lesions, tissue-specific microbiological analyses of ocular, intestinal, blood, liver, kidney, and spleen samples, strain homology analyses, and experimental validation to determine whether any pathogen-transmission link exists between intestinal and ocular lesions.

Culture-based isolation and 16S rRNA sequencing suggested that isolates assigned as putative *Vibrio harveyi* were frequently detected in the intestinal contents of the intestinal exposure group. Specifically, a total of 23 cultivable bacterial isolates were obtained from intestinal contents in the CT-CD-FB group and the CT-CD-JK group, including 11 isolates from the CT-CD-FB group and 12 isolates from the CT-CD-JK group. Identification results showed that isolates assigned as putative *Vibrio harveyi* accounted for 90.9% of the isolates from intestinal contents in the CT-CD-FB group, which was substantially higher than the 16.7% observed in the healthy control group. Thus, putative *Vibrio harveyi* was the dominant cultivable taxon in intestinal contents from the intestinal exposure group and may be associated with intestinal lesion development. Consistent with this finding, prior surveillance and reviews have indicated that *Vibrio harveyi* is frequently detected in mariculture vibriosis events and holds important epidemiological relevance [9]. In addition, the infection process of *Vibrio harveyi* may coincide with alterations in the host intestinal microbiological barrier, indicating a link between gut microbiota and host defense [10]. Regarding pathogenic mechanisms, recent comparative genomics and integrative studies have suggested pronounced strain-to-strain differences in *Vibrio harveyi* virulence, and differences in virulence-gene profiles and mobile genetic elements (e.g., plasmids) may influence pathogenic potential and host outcomes [11,12,18]. Accordingly, the higher detection proportion of putative *Vibrio harveyi* in the intestinal exposure group is consistent with a candidate-pathogen association but does not establish confirmed species identity or causality. Because this study used 16S rRNA for preliminary isolate identification, a single 16S sequence provides limited resolution for discriminating closely related species within the Harveyi clade. Thus, subsequent work should apply multilocus sequence typing or whole-genome sequencing to confirm taxonomic placement and should conduct experimental infection challenge to verify whether these isolates can induce intestinal exposure, intestinal pathological injury, or ocular lesions.

Antimicrobial susceptibility testing showed pronounced heterogeneity in the R/I/S profiles of the 23 isolates against 30 antibiotics, suggesting a potential multidrug-resistance risk. The isolates showed high R-category proportions for CB, P, E, AM, PIP, TE, CC, OX, and VA, whereas SXT showed an R-category proportion of 74%. A systematic review and meta-analysis of antimicrobial resistance in Asian aquatic animals has highlighted that resistance levels in bacteria derived from aquatic food animals warrant sustained attention and that resistance surveillance is important for both public health and aquaculture biosecurity. In parallel, studies on resistance in *Vibrio* spp. across aquatic environments have underscored the risks of resistance accumulation and dissemination and have called for systematic assessment and intervention within aquaculture management [17,19]. Because validated CLSI interpretive breakpoints are not available for all antimicrobial agents, bacterial taxa, and aquaculture-derived environmental isolates tested in this study, the present R/I/S categories should be interpreted cautiously. These results provide supportive in vitro information for antimicrobial-susceptibility assessment and candidate antimicrobial screening, but they should not be regarded as definitive predictions of clinical efficacy under farm conditions.

Overall, intestinal exposure and ocular opacity in hybrid porcupinefish were associated with gut microbiome imbalance, barrier injury, shifts in cultivable bacterial composition, and antimicrobial-resistance risk. Unlike studies limited to pathogen isolation or antimicrobial susceptibility testing alone, this study combined gut microbiota profiling, histopathology, bacterial isolation, and antimicrobial susceptibility testing to describe the pathological and microecological features of diseased fish. The results indicated that the intestinal exposure group not only showed reduced microbial richness and a shifted community structure but also exhibited a higher detection proportion of isolates assigned as putative *Vibrio harveyi* among cultivable bacteria in intestinal contents, suggesting that this bacterium is a candidate pathogen warranting focused attention. In addition, the ocular opacity group showed ocular histopathological injury together with altered intestinal community composition; however, these concurrent findings do not establish a direct gut–eye relationship, and current evidence remains insufficient to support a causal link with gut-derived putative *Vibrio harveyi*. Several limitations should be considered when interpreting these results. First, the sample size was limited to five fish per group, which restricts statistical power and limits the generalizability of the observed microbiome, pathological, and bacterial-isolation patterns. Second, the microbiome analysis was based on 97% OTU clustering rather than ASV-based inference. ASV approaches, such as DADA2, may provide higher sequence-level resolution and improve discrimination of closely related bacterial sequence variants. In addition, functional prediction, microbial co-occurrence or network analysis, and targeted validation of key taxa were not performed. Third, the available water-quality and aquaculture-management records provide sampling context but cannot prove or exclude the contribution of environmental fluctuations; turbidity was not measured. Therefore, the microbiome findings in this study should be interpreted mainly as descriptive and exploratory community-composition patterns rather than evidence of functional mechanisms or microbial interaction networks. Future studies should include larger sample sizes, ASV-based analysis, functional prediction or metagenomic sequencing, network analysis, and targeted validation of candidate taxa. These studies should also distinguish fish with intestinal exposure alone, ocular opacity alone, or both conditions, and collect intestinal, ocular, and major organ tissues for pathogen detection and homology analysis. Further experimental infection challenge could validate whether the isolated strains can reproduce intestinal injury or the ocular opacity phenotype. Genome sequencing and virulence gene-profile analyses could be used to characterize strain differences and evaluate their potential pathogenicity. At the farm level, water-quality monitoring, feed management, rapid pathogen detection, and antimicrobial susceptibility testing may help reduce reliance on empirical antibiotic use. Together, these findings provide preliminary descriptive information for interpreting microecological and pathological features associated with intestinal exposure and ocular opacity and for guiding subsequent targeted pathogen-verification and antimicrobial-susceptibility studies.

## 5. Conclusions

This study indicates that intestinal exposure and ocular opacity in hybrid porcupinefish were associated with gut microecological imbalance, tissue injury, and changes in cultivable bacterial composition in this small cohort of naturally diseased fish. Compared with the healthy control group, the intestinal exposure group showed reduced gut microbial richness. The three groups also differed in community structure, and phylum-level and differential-taxa analyses showed changes in dominant taxa and group-specific enrichment patterns in diseased fish. Histopathology showed disruption of the intestinal wall structure, villus atrophy or fusion, epithelial sloughing, edema, and inflammatory cell infiltration in diseased fish, whereas individuals with ocular opacity also exhibited swelling of the eyeball wall, disorganized tissue stratification, and periocular edema. Culture-based isolation and 16S rRNA sequence alignment further showed that isolates assigned as putative *Vibrio harveyi* accounted for a higher proportion among isolates from intestinal contents in the CT-CD-FB group than in the healthy control group, suggesting a candidate-pathogen association with intestinal exposure. However, this taxonomic assignment and its pathogenic role require further confirmation through species-specific marker-gene sequencing, such as *gyrB*, *toxR*, or *rpoD*, whole-genome sequencing, experimental infection challenge, strain homology analysis, and multi-tissue pathogen detection. Antimicrobial susceptibility testing showed larger proportions of R-category results for several tested antimicrobials, whereas larger proportions of S-category results were observed for CTR, some quinolones, aminoglycosides, and chloramphenicol. These preliminary findings may provide reference information for subsequent pathogen-verification studies and antimicrobial-susceptibility assessment in hybrid porcupinefish aquaculture. In summary, this study combines gut microbiota profiling, histopathology, bacterial isolation, and antimicrobial susceptibility testing to describe microecological and pathological features associated with intestinal exposure and ocular opacity in hybrid porcupinefish.

## Figures and Tables

**Table 1:** Experimental Groups and Sample Uses.

Group	Abbreviation	Clinical Characteristics	Sample Size	Sample Type	Main Use
Healthy control group	CT-CD-JK	No obvious clinical symptoms	n = 5	Intestinal contents; tissue samples	16S sequencing; bacterial isolation; pathological observation
Intestinal exposure group	CT-CD-FB	Intestinal/rectal prolapse	n = 5	Intestinal contents; tissue samples	16S sequencing; bacterial isolation; pathological observation
Ocular opacity group	CT-YQ-FB	Ocular opacity; corneal or lens abnormalities	n = 5	Intestinal contents; ocular tissue	16S sequencing; bacterial isolation; pathological observation

**Table 2:** Quantitative Histopathological Indicators of Intestinal Tissues in Three Pufferfish Groups.

Indicator	Unit	CT-CD-JK	CT-CD-FB	CT-YQ-FB	*p* Value
Intestinal villus height	μm	520.4 ± 58.7 ^a^	286.3 ± 46.5 ^c^	412.8 ± 63.2 ^b^	<0.01
Intestinal villus width	μm	82.6 ± 10.8 ^c^	146.7 ± 22.4 ^a^	108.5 ± 17.9 ^b^	<0.01
Intestinal wall thickness	μm	185.3 ± 24.6 ^c^	318.9 ± 41.7 ^a^	236.4 ± 32.8 ^b^	<0.01
Mucosal layer thickness	μm	148.2 ± 19.5 ^a^	92.7 ± 16.3 ^c^	126.5 ± 21.4 ^b^	<0.05
Number of goblet cells	cells/field	18.4 ± 3.2 ^c^	34.6 ± 5.8 ^a^	25.7 ± 4.6 ^b^	<0.01
Epithelial shedding score	0–3 points	0.4 ± 0.5 ^c^	2.7 ± 0.5 ^a^	1.5 ± 0.5 ^b^	<0.01
Inflammatory cell infiltration score	0–3 points	0.6 ± 0.5 ^c^	2.8 ± 0.4 ^a^	1.7 ± 0.5 ^b^	<0.01
Lamina propria edema score	0–3 points	0.3 ± 0.5 ^c^	2.5 ± 0.5 ^a^	1.4 ± 0.5 ^b^	<0.01
Total intestinal pathological injury score	points	1.3 ± 0.9 ^c^	8.0 ± 1.1 ^a^	4.6 ± 1.2 ^b^	<0.01

Note: Data are presented as mean ± SD. CT-CD-JK: healthy control group; CT-CD-FB: intestinal exposure group; CT-YQ-FB: Ocular opacity group. Different lowercase letters indicate significant differences among groups, *p* < 0.05. Epithelial shedding, inflammatory cell infiltration, and lamina propria edema were scored semi-quantitatively on a 0–3 scale: 0, no obvious lesion; 1, mild; 2, moderate; 3, severe. The total intestinal pathological injury score is the sum of the epithelial shedding score, inflammatory cell infiltration score, and lamina propria edema score.

**Table 3:** PCoA and PERMANOVA Analysis of Differences in Intestinal Microbiota Structure among the Three Groups.

Analysis Item	Metric	Result
PCoA	PC1 explained variance	36%
PCoA	PC2 explained variance	18%
PCoA	PC1 + PC2 total explained variance	54%
PERMANOVA	df	2
PERMANOVA	R^2^	0.5058
PERMANOVA	*p* value	0.001
Intergroup distance	Main result	The distance between CT-CD-JK and the diseased groups was relatively large, whereas CT-CD-FB and CT-YQ-FB were relatively close.

Note: Compiled based on the PCoA and PERMANOVA results. PERMANOVA was performed based on the community distance matrix with 999 permutations.

**Table 4:** Summary of 16S rRNA Molecular Identification Results for Culturable Bacterial Isolates.

Identification Result	No. of Isolates from CT-CD-FB Intestinal Contents	No. of Isolates from CT-CD-JK Intestinal Contents	Total Isolates	Main Distribution Pattern
*Vibrio harveyi*	10	2	12	Highest proportion among isolates from CT-CD-FB intestinal contents
*Bacterium 4H202*	1	0	1	Only observed among isolates from CT-CD-FB intestinal contents
*Bacterium 4H104*	0	1	1	Only observed among isolates from CT-CD-JK intestinal contents
*Vibrio campbellii*	0	3	3	Detected among isolates from CT-CD-JK intestinal contents
*Vibrio tubiashii*	0	1	1	Detected among isolates from CT-CD-JK intestinal contents
*Vibrio* sp. *NTOU-M3*	0	1	1	Detected among isolates from CT-CD-JK intestinal contents
*Vibrio alginolyticus*	0	1	1	Detected among isolates from CT-CD-JK intestinal contents
*Vibrio parahaemolyticus*	0	2	2	Detected among isolates from CT-CD-JK intestinal contents
*Vibrio* sp. *JPW-8-11-4*	0	1	1	Detected among isolates from CT-CD-JK intestinal contents

**Table 5:** Summary of Antimicrobial Susceptibility Results of 23 Isolates against 30 Antibiotics.

Antibiotic	Abbreviation	Summary of Antimicrobial Susceptibility Results
Cefoperazone	CFP	The isolates showed variable R/I/S profiles
Carbenicillin	CB	R-category proportion > 80%
Penicillin G	P	R-category proportion > 80%
Furazolidone	FZ	R-category proportion < 20%; higher proportion of susceptible isolates
Polymyxin B	PB	The isolates showed variable R/I/S profiles
Erythromycin	E	R-category proportion > 80%
Sulfamethoxazole/trimethoprim	SXT	R-category proportion 74%
Ampicillin	AM	R-category proportion > 80%
Ofloxacin	OFX	R-category proportion < 20%; higher proportion of susceptible isolates
Piperacillin	PIP	R-category proportion > 80%
Kanamycin	K	R-category proportion < 20%; higher proportion of susceptible isolates
Ceftriaxone	CTR	S-category proportion 91%
Ceftazidime	CAZ	R-category proportion < 20%; higher proportion of susceptible isolates
Tetracycline	TE	R-category proportion > 80%
Doxycycline	DX	The isolates showed variable R/I/S profiles
Gentamicin	GM	R-category proportion < 20%; higher proportion of susceptible isolates
Norfloxacin	NOR	R-category proportion < 20%; higher proportion of susceptible isolates
Clindamycin	CC	R-category proportion > 80%
Ciprofloxacin	CIP	R-category proportion < 20%; higher proportion of susceptible isolates
Oxacillin	OX	R-category proportion > 80%
Cefuroxime	CXM	R-category proportion < 20%; higher proportion of susceptible isolates
Cephradine	RAD	The isolates showed variable R/I/S profiles
Midecamycin	MD	The isolates showed variable R/I/S profiles
Cefazolin	CZ	R-category proportion < 20%; higher proportion of susceptible isolates
Minocycline	MI	The isolates showed variable R/I/S profiles
Chloramphenicol	C	R-category proportion < 20%; higher proportion of susceptible isolates
Amikacin	AK	R-category proportion < 20%; higher proportion of susceptible isolates
Vancomycin	VA	R-category proportion > 80%
Cephalexin	CA	The isolates showed variable R/I/S profiles
Neomycin	N	R-category proportion < 20%; higher proportion of susceptible isolates

Note: Compiled based on the antimicrobial susceptibility results of 23 isolates against 30 antibiotics. R: resistant; I: intermediate; S: susceptible.

## Data Availability

The raw PacBio 16S rRNA amplicon sequencing data generated in this study have been deposited in the NCBI Sequence Read Archive under BioProject accession number PRJNA1478811. The SRA accession numbers are SRR39175234-SRR39175248, including CT-CD-JK samples SRR39175234-SRR39175238, CT-CD-FB samples SRR39175239-SRR39175241, SRR39175247, and SRR39175248, and CT-YQ-FB samples SRR39175242-SRR39175246.. Sample-level BioProject and SRA accession information is provided in Table S3. Because separate GenBank accession numbers for the cultivable-isolate 16S rRNA gene sequences were not available at the time of this revision, unconfirmed GenBank identifiers were not reported. The isolate-level 16S rRNA identification results are provided in Table S5, and per-sample sequencing reads and FASTQ-derived QC metrics are provided in Table S2. Other data generated or analyzed during this study are included in this article and its Supplementary Material files. Further inquiries can be directed to the corresponding author.

## Supplementary Materials

The following supporting information can be downloaded at: https://www.mdpi.com/article/10.3390/microorganisms14081703/s1, Figure S1: Supplementary analysis of intestinal microbiota α diversity; Figure S2: Analysis of core microbiota and shared microbiota among three groups of samples; Figure S3: Supplementary analysis of gut microbiota β-diversity in three groups of samples; Figure S4: Gut microbiota composition at different taxonomic levels and distribution characteristics of dominant phyla across three groups of samples; Table S1: Information on the 30 Antibiotic Susceptibility Discs Used in This Study; Table S2: Per-sample sequencing reads and FASTQ-derived quality-control metrics; Table S3: NCBI BioProject and SRA Accession Numbers for PacBio 16S rRNA Sequencing Samples; Table S4: Major Differential Taxa Identified by LEfSe Analysis; Table S5: Detailed 16S rRNA-Based Identification Results for 23 Culturable Isolates.

## Abbreviations

AK	Amikacin
AM	Ampicillin
ANOVA	Analysis of Variance
C	Chloramphenicol
CA	Cephalexin
CAZ	Ceftazidime
CB	Carbenicillin
CC	Clindamycin
CFP	Cefoperazone
CFU	Colony-Forming Unit
CIP	Ciprofloxacin
CLSI	Clinical and Laboratory Standards Institute
CTR	Ceftriaxone
CXM	Cefuroxime
CZ	Cefazolin
DX	Doxycycline
E	Erythromycin
FDR	False Discovery Rate
FZ	Furazolidone
GM	Gentamicin
H&E	Hematoxylin and Eosin
I	Intermediate
K	Kanamycin
LDA	Linear Discriminant Analysis
LEfSe	Linear Discriminant Analysis Effect Size
MD	Midecamycin
MI	Minocycline
N	Neomycin
NMDS	Non-Metric Multidimensional Scaling
NOR	Norfloxacin
OFX	Ofloxacin
OTUs	Operational Taxonomic Units
OX	Oxacillin
P	Penicillin G
PB	Polymyxin B
PCA	Principal Component Analysis
PCoA	Principal Coordinates Analysis
PCR	Polymerase Chain Reaction
PERMANOVA	Permutational Multivariate Analysis of Variance
PIP	Piperacillin
R	Resistant
RAD	Cephradine
S	Susceptible
SD	Standard Deviation
SXT	Sulfamethoxazole-Trimethoprim
TE	Tetracycline
UPGMA	Unweighted Pair Group Method with Arithmetic Mean
VA	Vancomycin
