# Supplementary material for: Intestinal Microecology Dysbiosis, Cultivable Pathogen Composition, and Antimicrobial Susceptibility Profiles Associated with Intestinal Exposure and Ocular Opacity in Hybrid Porcupinefish
Source: Microorganisms. 2026 Aug 3;14(8):1703. doi: 10.3390/microorganisms14081703 (PMC13515036; doi:10.3390/microorganisms14081703)
Supplement: Supplementary file 1 [file microorganisms-14-01703-s001.zip › microorganisms-4358445-supplementary.pdf]

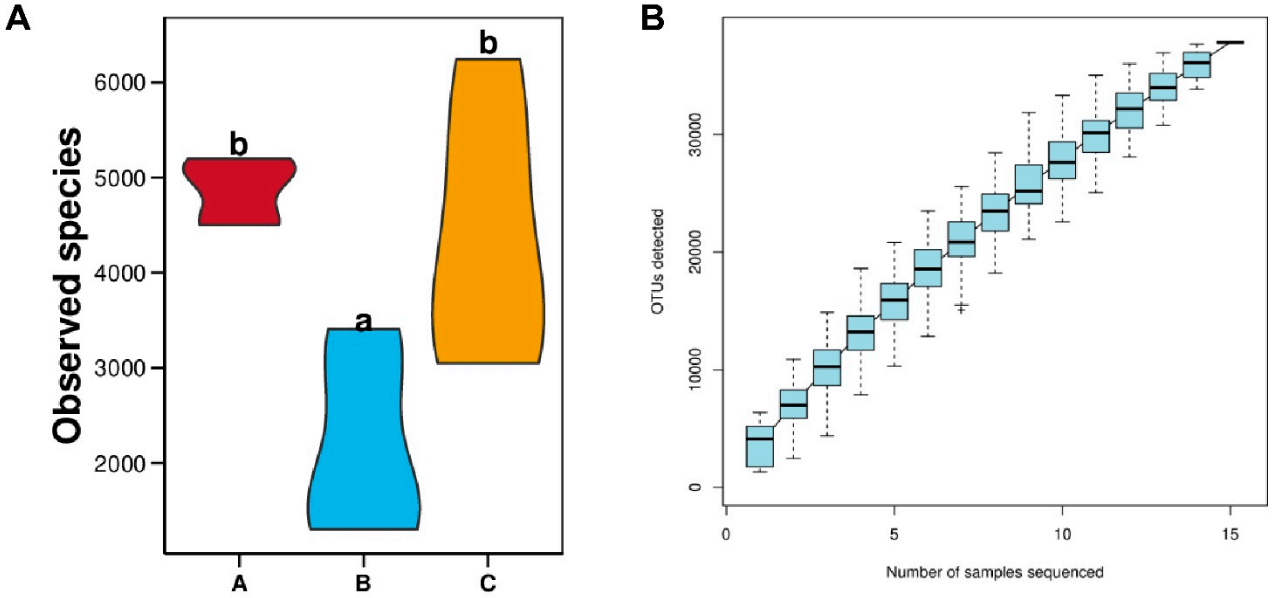

Figure S1. Supplementary analysis of intestinal microbiota α diversity. (A) Violin plot of Observed species for the three groups of samples. The A, B, and C on the x-axis represent the CT-CD-JK group (healthy control), CT-CD-FB group (intestinal exposure), and CT-YQ-FB group (corneal opacity), respectively. Different lowercase letters indicate significant differences between groups (p < 0.05), while identical letters indicate non-significant differences (p > 0.05). (B) OTU accumulation boxplot, showing the increasing trend of cumulative OTUs with the increase in the number of sequenced samples included. CT-CD-JK: healthy control; CT-CD-FB: intestinal exposure; CT-YQ-FB: corneal opacity.

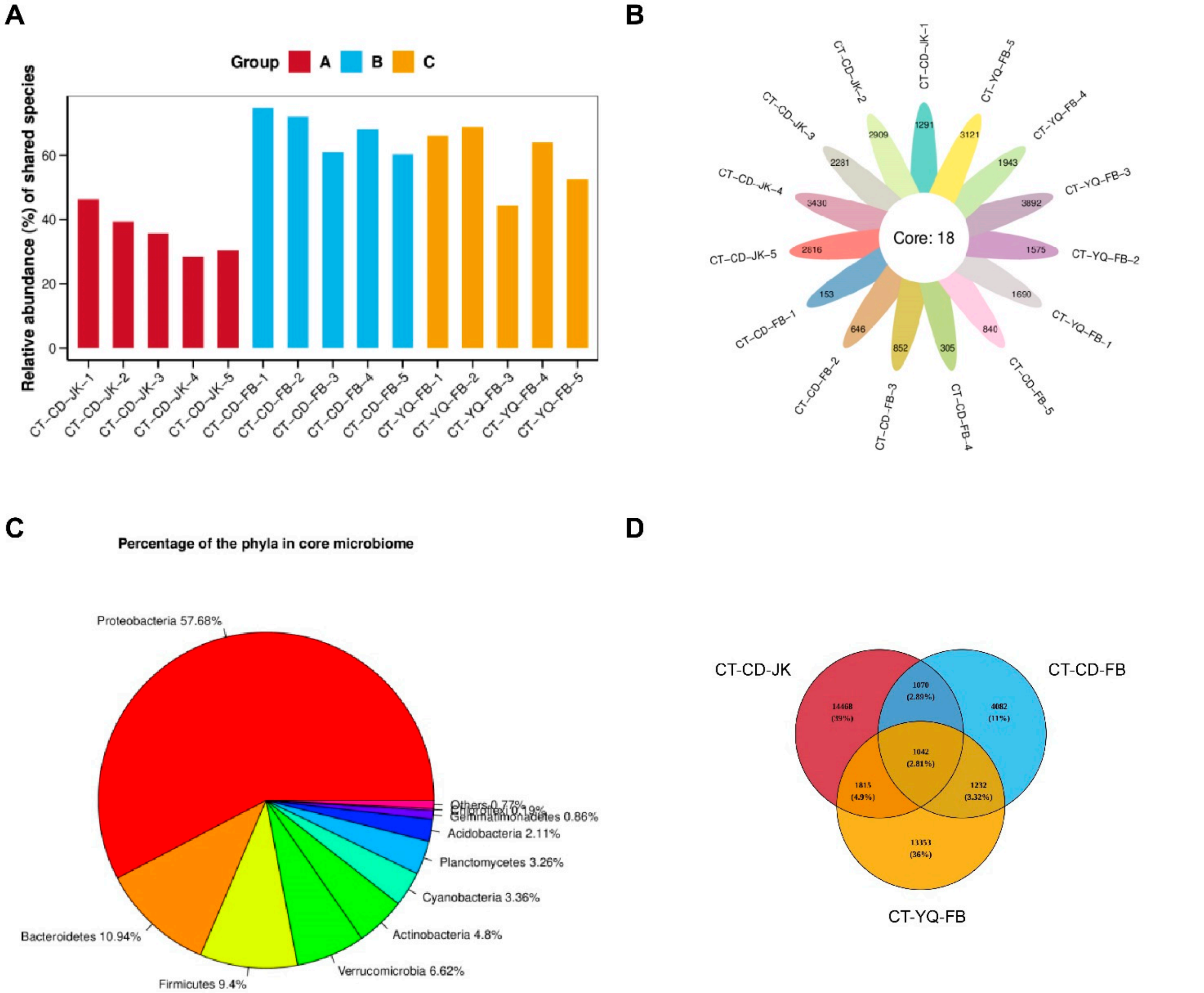

Figure S2. Analysis of core microbiota and shared microbiota among three groups of samples. (A) Bar chart of the relative abundance of shared species among the three groups; A, B, and C represent the CT-CD-JK group, CT-CD-FB group, and CT-YQ-FB group, respectively. (B) Petal plot of core microbiota and unique microbiota in the samples; the central number indicates the number of core microbial taxa shared by all samples, and the numbers on the petals indicate the number of unique microbial taxa in each individual sample. (C) Percentage composition of core microbiota at the phylum level, showing the proportional composition of core microbiota shared by all samples at the phylum level. (D) Venn diagram of microbial taxa among the three groups; overlapping regions indicate the number of microbial taxa shared between groups, and non-overlapping regions indicate the number of microbial taxa unique to each group. CT-CD-JK: healthy control group; CT-CD-FB: Intestinal exposure group; CT-YQ-FB: Ocular opacity group.

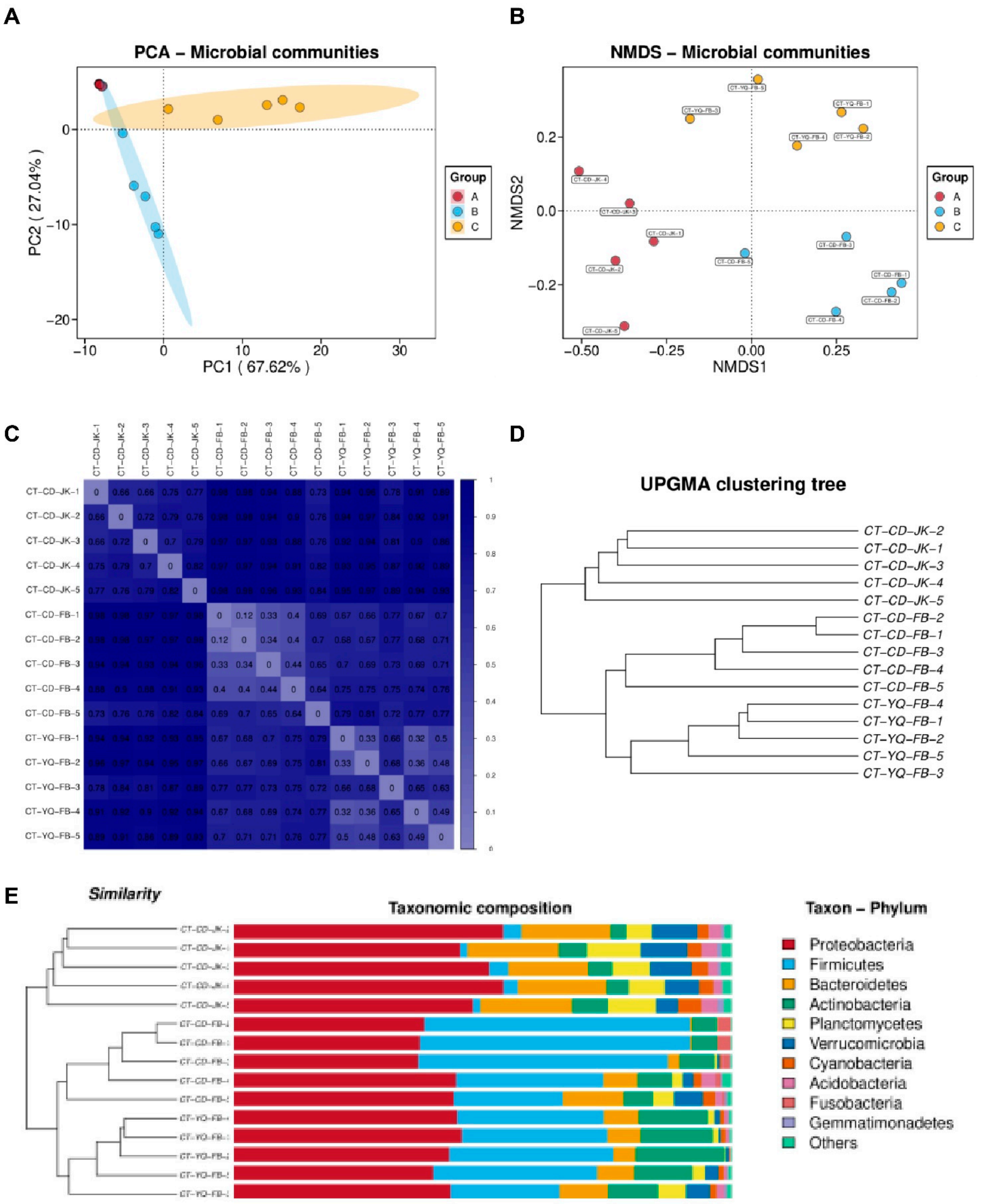

Figure S3. Supplementary analysis of gut microbiota β-diversity in three groups of samples. (A) PCA plot; (B) NMDS plot; (C) Heatmap of community similarity among samples; (D) UPGMA clustering tree; (E) Cluster analysis of sample community similarity and phylum-level composition. CT-CD-JK: healthy control group; CT-CD-FB: intestinal exposure group; CT-YQ-FB: ocular opacity group.

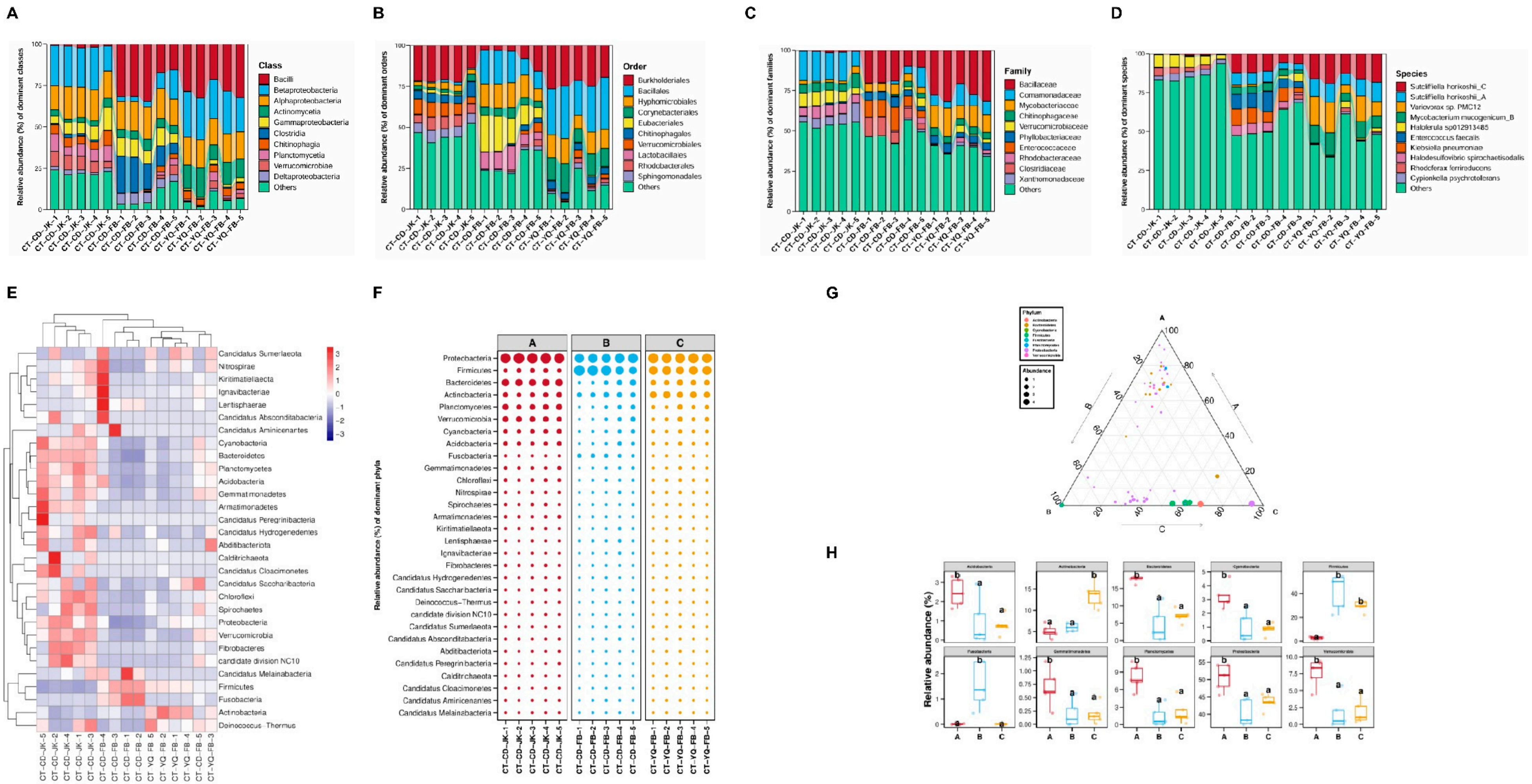

Figure S4. Gut microbiota composition at different taxonomic levels and distribution characteristics of dominant phyla across three groups of samples. (A) Stacked bar plot of the relative abundance of microbial communities at the class level; (B) stacked bar plot of the relative abundance of microbial communities at the order level; (C) stacked bar plot of the relative abundance of microbial communities at the family level; (D) stacked bar plot of the relative abundance of microbial communities at the species level; (E) heatmap of the relative

abundance of microbial communities at the phylum level, with colors ranging from blue to red indicating low to high relative abundance; (F) bubble plot of the relative abundance of microbial communities at the phylum level, with bubble size indicating relative abundance; (G) ternary plot of dominant phyla, showing the distribution of major dominant phyla among the three groups; (H) boxplot of the relative abundance of dominant phyla, comparing abundance differences of major dominant phyla among groups. In the figure, groups A, B, and C represent the CT-CD-JK group, CT-CD-FB group, and CT-YQ-FB group, respectively. CT-CD-JK: healthy control group; CT-CD-FB: intestinal exposure group; CT-YQ-FB: ocular opacity group. In panel H, different lowercase letters indicate significant differences among groups ($p < 0.05$), whereas the same letter indicates no significant difference ($p > 0.05$).

Table S1. Information on the 30 Antibiotic Susceptibility Discs Used in This Study

No.	Chinese name	English name	Abbreviation used in this paper	Susceptibility disc code	Disc content	Manufacturer	Catalog No.
1	Cefoperazone	Cefoperazone	CFP	CFP	75 μg/disc	Thermo Scientific Oxoid	CT0249B
2	Carbenicillin	Carbenicillin	CB	CAR	100 μg/disc	Thermo Scientific Oxoid	CT0006B
3	Penicillin G	Penicillin G	P	P	10 U/disc	Thermo Scientific Oxoid	CT0043B
4	Furazolidone	Furazolidone	FZ	FR	100 μg/disc	Thermo Scientific Oxoid	CT0122B
5	Polymyxin B	Polymyxin B	PB	PB	300 U/disc	Thermo Scientific Oxoid	CT0044B
6	Erythromycin	Erythromycin	E	E	15 μg/disc	Thermo Scientific Oxoid	CT0020B
7	Trimethoprim/Sulfa methoxazole	Trimethoprim/Sulfa methoxazole	SXT	SXT	1.25/23.75 μg/disc; total 25 μg/disc	Thermo Scientific Oxoid	CT0052B
8	Ampicillin	Ampicillin	AM	AMP	10 μg/disc	Thermo Scientific Oxoid	CT0003B
9	Ofloxacin	Ofloxacin	OFX	OFX	5 μg/disc	Thermo Scientific Oxoid	CT0446B
10	Piperacillin	Piperacillin	PIP	PRL	100 μg/disc	Thermo Scientific Oxoid	CT0199B
11	Kanamycin	Kanamycin	K	K	30 μg/disc	Thermo Scientific Oxoid	CT0026B
12	Ceftriaxone	Ceftriaxone	CTR	CRO	30 μg/disc	Thermo Scientific Oxoid	CT0417B
13	Ceftazidime	Ceftazidime	CAZ	CAZ	30 μg/disc	Thermo Scientific Oxoid	CT0412B
14	Tetracycline	Tetracycline	TE	TE	30 μg/disc	Thermo Scientific Oxoid	CT0054B
15	Doxycycline	Doxycycline	DX	DO	30 μg/disc	Thermo Scientific Oxoid	CT0018B
16	Gentamicin	Gentamicin	GM	CN	10 μg/disc	Thermo Scientific Oxoid	CT0024B
17	Norfloxacin	Norfloxacin	NOR	NOR	10 μg/disc	Thermo Scientific Oxoid	CT0434B
18	Clindamycin	Clindamycin	CC	DA	2 μg/disc	Thermo Scientific Oxoid	CT0064B
19	Ciprofloxacin	Ciprofloxacin	CIP	CIP	5 μg/disc	Thermo Scientific Oxoid	CT0425B
20	Oxacillin	Oxacillin	OX	OX	1 μg/disc	Thermo Scientific Oxoid	CT0159B
21	Cefuroxime sodium	Cefuroxime sodium	CXM	CXM	30 μg/disc	Thermo Scientific Oxoid	CT0127B
22	Cephradine	Cephradine	RAD	CE	30 μg/disc	Thermo Scientific Oxoid	CT0063B
23	Midecamycin	Midecamycin	MD	MD	30 μg/disc	Hangzhou Microbial Reagents/OWD series	OWD-104 5
24	Cefazolin/Cephazoli n	Cefazolin/Cephazoli n	CZ	KZ	30 μg/disc	Thermo Scientific Oxoid	CT0011B
25	Minocycline	Minocycline	MI	MH	30 μg/disc	Thermo Scientific Oxoid	CT0030B
26	Chloramphenicol	Chloramphenicol	C	C	30 μg/disc	Thermo Scientific Oxoid	CT0013B

No.	Chinese name	English name	Abbreviation used in this paper	Susceptibility disc code	Disc content	Manufacturer	Catalog No.
27	Amikacin	Amikacin	AK	AK	30 μg/disc	Thermo Scientific Oxoid	CT0107B
28	Vancomycin	Vancomycin	VA	VA	30 μg/disc	Thermo Scientific Oxoid	CT0058B
29	Cephalexin	Cephalexin	CA	CL	30 μg/disc	Thermo Scientific Oxoid	CT0007B
30	Neomycin	Neomycin	N	N	30 μg/disc	Thermo Scientific Oxoid	CT0033B

Table S2. Per-sample sequencing reads and FASTQ-derived quality-control metrics.

Sample	Report sequences	FASTQ reads	Delta	Total bases	Mean length (bp)	Median length (bp)	Q20 (%)	Q30 (%)	GC (%)	N (%)
CT-CD-FB-1	32988	32988	0	47886194	1451.62	1454	98.63	96.90	54.16	0.0000
CT-CD-FB-2	30800	30800	0	44702773	1451.39	1454	98.74	97.15	54.18	0.0000
CT-CD-FB-3	40106	40106	0	58124226	1449.27	1453	98.61	96.87	54.31	0.0000
CT-CD-FB-4	35051	35051	0	50765013	1448.32	1452	98.65	96.94	54.34	0.0000
CT-CD-FB-5	36463	36463	0	52719578	1445.84	1452	98.62	96.89	54.17	0.0000
CT-CD-JK-1	37507	37507	0	54126387	1443.10	1450	98.60	96.84	54.38	0.0000
CT-CD-JK-2	34320	34320	0	49510513	1442.61	1450	98.82	97.35	54.00	0.0000
CT-CD-JK-3	30181	30181	0	43513468	1441.75	1450	98.68	97.03	54.37	0.0000
CT-CD-JK-4	32253	32253	0	46490722	1441.44	1449	98.86	97.44	54.18	0.0000
CT-CD-JK-5	31073	31073	0	44768334	1440.75	1446	98.77	97.23	54.05	0.0000
CT-YQ-FB-1	33661	33661	0	48771408	1448.90	1456	98.91	97.55	55.07	0.0000
CT-YQ-FB-2	33039	33039	0	47892814	1449.58	1456	98.83	97.37	55.32	0.0000
CT-YQ-FB-3	32488	32488	0	47023658	1447.42	1454	98.93	97.58	54.74	0.0000
CT-YQ-FB-4	33713	33713	0	48843007	1448.79	1456	98.86	97.42	55.03	0.0000
CT-YQ-FB-5	32774	32774	0	47485765	1448.89	1456	98.86	97.43	55.00	0.0000

Note: The raw 16S rRNA amplicon sequencing data have been deposited in the NCBI Sequence Read Archive under BioProject accession number PRJNA1478811. Sample-level SRA accession numbers are provided in Supplementary Table S3. PacBio 16S rRNA amplicon sequencing targeted the V1-V9 region using primers 27F/1492R, with 27 PCR cycles.

Table S3. NCBI BioProject and SRA Accession Numbers for PacBio 16S rRNA Sequencing Samples

Group	Sample	SRA accession no.	BioProject accession no.	Target region	Primer pair	PCR cycles
CT-CD-FB	CT-CD-FB-1	SRR39175248	PRJNA1478811	V1-V9	27F/1492R	27
CT-CD-FB	CT-CD-FB-2	SRR39175247	PRJNA1478811	V1-V9	27F/1492R	27
CT-CD-FB	CT-CD-FB-3	SRR39175241	PRJNA1478811	V1-V9	27F/1492R	27
CT-CD-FB	CT-CD-FB-4	SRR39175240	PRJNA1478811	V1-V9	27F/1492R	27
CT-CD-FB	CT-CD-FB-5	SRR39175239	PRJNA1478811	V1-V9	27F/1492R	27
CT-CD-JK	CT-CD-JK-1	SRR39175238	PRJNA1478811	V1-V9	27F/1492R	27
CT-CD-JK	CT-CD-JK-2	SRR39175237	PRJNA1478811	V1-V9	27F/1492R	27
CT-CD-JK	CT-CD-JK-3	SRR39175236	PRJNA1478811	V1-V9	27F/1492R	27
CT-CD-JK	CT-CD-JK-4	SRR39175235	PRJNA1478811	V1-V9	27F/1492R	27
CT-CD-JK	CT-CD-JK-5	SRR39175234	PRJNA1478811	V1-V9	27F/1492R	27
CT-YQ-FB	CT-YQ-FB-1	SRR39175246	PRJNA1478811	V1-V9	27F/1492R	27
CT-YQ-FB	CT-YQ-FB-2	SRR39175245	PRJNA1478811	V1-V9	27F/1492R	27
CT-YQ-FB	CT-YQ-FB-3	SRR39175244	PRJNA1478811	V1-V9	27F/1492R	27
CT-YQ-FB	CT-YQ-FB-4	SRR39175243	PRJNA1478811	V1-V9	27F/1492R	27
CT-YQ-FB	CT-YQ-FB-5	SRR39175242	PRJNA1478811	V1-V9	27F/1492R	27

Note: The NCBI metadata grouping corresponds to control = CT-CD-JK, case1 = CT-CD-FB, and case2 = CT-YQ-FB. The BioProject/SRA submission status was shown as processed in NCBI at the time of revision.

Table S4. Major Differential Taxa Identified by LEfSe Analysis

Enriched group	Major differential taxonomic units	Main taxonomic branches	Interpretation
CT-CD-JK	Sphingobacteriaceae, Cytophagaceae, Haliscomenobacteraceae	Bacteroidetes-related branches	Bacteroidetes-related taxa were enriched in the healthy control group
CT-CD-FB	Fusobacteriaceae, Clostridiaceae, Lactobacillaceae	Firmicutes- and Fusobacteria-related branches	Firmicutes- and Fusobacteria-related taxa were enriched in the intestinal prolapse group
CT-YQ-FB	Campylobacteraceae, Oceanospirillaceae, Vibrionaceae	Proteobacteria-related branches	Proteobacteria-related taxa were enriched in the eye opacity group

Table S5. Detailed 16S rRNA-Based Identification Results for 23 Culturable Isolates

Isolate ID	Source group	Sample source	Identification result
B01	CT-CD-FB	Intestinal contents	*Vibrio harveyi*
B03	CT-CD-FB	Intestinal contents	*Vibrio harveyi*
B04	CT-CD-FB	Intestinal contents	*Vibrio harveyi*
B05	CT-CD-FB	Intestinal contents	*Vibrio harveyi*
B07	CT-CD-FB	Intestinal contents	*Vibrio harveyi*
B09	CT-CD-FB	Intestinal contents	*Vibrio harveyi*
B10	CT-CD-FB	Intestinal contents	Bacterium 4H202
B11	CT-CD-FB	Intestinal contents	*Vibrio harveyi*
B12	CT-CD-FB	Intestinal contents	*Vibrio harveyi*
B13	CT-CD-FB	Intestinal contents	*Vibrio harveyi*
B14	CT-CD-FB	Intestinal contents	*Vibrio harveyi*
J01	CT-CD-JK	Intestinal contents	Bacterium 4H104
J03	CT-CD-JK	Intestinal contents	*Vibrio campbellii*
J04	CT-CD-JK	Intestinal contents	*Vibrio tubiashii*
J05	CT-CD-JK	Intestinal contents	*Vibrio sp. NTOU-M3*
J06	CT-CD-JK	Intestinal contents	*Vibrio campbellii*
J07	CT-CD-JK	Intestinal contents	*Vibrio alginolyticus*
J08	CT-CD-JK	Intestinal contents	*Vibrio parahaemolyticus*
J09	CT-CD-JK	Intestinal contents	*Vibrio campbellii*
J10	CT-CD-JK	Intestinal contents	*Vibrio parahaemolyticus*
J11	CT-CD-JK	Intestinal contents	*Vibrio harveyi*
J12	CT-CD-JK	Intestinal contents	*Vibrio harveyi*
J13	CT-CD-JK	Intestinal contents	*Vibrio sp. JPW-8-11-4*

Note: Intestinal contents. The identification results are based on 16S rRNA sequence alignment. Species-level assignments within the genus Vibrio should be interpreted as putative because gyrB, toxR, rpoD, multilocus sequence analysis, and whole-genome sequencing were not performed in this study.
